# Cross-modal impacts of olfactory perception and individual differences in emotional responses to odors modulate hedonic evaluations of visual scenes

**DOI:** 10.1093/chemse/bjag027

**Published:** 2026-08-25

**Authors:** Lucrezia Pacinotti, Carl Roberts, Timo Giesbrecht, Nicholas Fallon

**Affiliations:** Department of Psychology, Institute of Population Health, Faculty of Health and Life Sciences, University of Liverpool, Eleanor Rathbone building, Bedford Street South, Liverpool L69 7ZA, United Kingdom; Department of Psychology, Institute of Population Health, Faculty of Health and Life Sciences, University of Liverpool, Eleanor Rathbone building, Bedford Street South, Liverpool L69 7ZA, United Kingdom; Department of Psychology, Institute of Population Health, Faculty of Health and Life Sciences, University of Liverpool, Eleanor Rathbone building, Bedford Street South, Liverpool L69 7ZA, United Kingdom; Unilever Research & Development, Personal Care, Quarry Rd E, Port Sunlight CH63 3JW, United Kingdom; Department of Psychology, Institute of Population Health, Faculty of Health and Life Sciences, University of Liverpool, Eleanor Rathbone building, Bedford Street South, Liverpool L69 7ZA, United Kingdom

**Keywords:** olfaction, odor–visual, multisensory integration, human, emotions

## Abstract

In line with the critical role of human olfaction in motivated behavior, positively valenced odors have been shown to boost hedonic perception of visual stimuli, especially facial expressions, compared to unpleasant smells. However, it remains unclear whether pleasant fragrances, which vary in character and subjective affective profile, differentially impact different categories of visual stimuli. We also postulate that individual differences in emotional responses to odors modulate their subsequent effect on hedonic evaluations of images. The current study investigated how (relative to a clean air control) 4 pleasant odors (vanilla, rose, lavender, and lemon) influenced hedonic ratings of different categories of visual images (faces, landscapes, and objects), with each category comprising images of neutral and positive valence. Forty participants (aged 18 to 35) rated the pleasantness of each image during each odor condition presented using a computer-controlled olfactometer. Participants also reported their emotional response to each fragrance on 7 dimensions (happiness, disgust, relaxation, thirstiness, sensuality, energy, and nostalgia) using the Liverpool Emotion and Odor Scale (LEOS), self-report questionnaire. Pleasant odors significantly affected image pleasantness ratings, but the effects of specific fragrances differed by image category. Individual differences in emotional experiences elicited by each fragrance were shown to modulate the impact of odors on hedonic evaluations, and these effects varies across the different image categories. The findings offer new insights into the differential effects of pleasant odors on hedonic evaluations of visual stimuli and the role of individual affective processing in cross-modal olfactory-visual integration.

## Introduction

Human olfaction plays a critical role in everyday life, influencing behavior both consciously and unconsciously, for instance by inducing avoidance when smelling something unpleasant (Bratman et al. 2024; Dikeçligil and Gottfried 2024). While breathing, our nose constantly informs us about the environment, facilitating rapid decision-making (Olofsson 2014). This is supported by olfaction's unique neural pathways. Unlike other sensory systems, olfactory signals bypass the thalamocortical relay, which is involved in modulatory and mediatory roles in cognitive processing. Instead, these signals connect directly to limbic regions responsible for emotions and cognition (Tham et al. 2009; Bratman et al. 2024).

Evidence suggests that vision and olfaction interact bidirectionally, especially when cues are congruent in hedonics; for example, visual stimuli can enhance odor pleasantness, and odors can enhance the perceived pleasantness of an image (Pollatos et al. 2007; Amsellem et al. 2018). Behavioral studies show that odors influence face evaluation: for example, pleasant odors can enhance facial attractiveness and improve expression recognition, whereas unpleasant odors tend to reduce attractiveness ratings and negatively bias the perception of the facial expressions (Demattè et al. 2007; Chen and Spence 2022). Despite some inconsistent findings, a review by Syrjänen et al. (2021) concludes that odors influence face perception in the direction of their valence; for instance, pleasant fragrances generally increase positive evaluations. However, notable heterogeneity exists across studies, partly because individual differences in sensory perception, context and cognition are not consistently considered (Syrjänen et al. 2021).

Most of the research in this area has focused on faces as visual stimuli, although some studies have explored how odors influence emotional judgments of non-human images, such as animals, landscapes or objects. These studies have shown that odors can affect emotional ratings of such images, ranging from unpleasant pictures (e.g. insects, mutilation, unhygienic/dirty scenes, and frightening animals) to more pleasant images (e.g. flowers) and erotic content (Walla and Deecke 2010; Adolph and Pause 2012). A study by Jiang et al. (2016) found no effects of odors on participants’ hedonic and quality evaluations of landscapes, using images that varied in traffic levels (low, medium, high, and none as control) and tree density. However, they observed that odors might modulate perception of landscapes in relation to specific environmental features (Jiang et al. 2016). This indicates that further research on non-human images is needed to clarify how odors influence the perception of visual stimuli and to determine whether cross-modal olfactory effects generalize beyond faces.

Cross-cultural research shows that hedonic responses to odors are related to how frequently an individual encounters a specific odor in one or more life phases and how that odor is perceived within their society (Delplanque et al. 2008; Ferdenzi et al. 2011; Barbosa et al. 2024). As a result, odors may elicit different emotional responses depending on the cultural background in which an odor is perceived. To address this, Ferdenzi et al. (2013) developed a series of culture-specific self-report scales, such as the Liverpool Emotion and Odor Scale (LEOS) and the Geneva Emotion and Odor Scale (GEOS), to measure subjective levels of affective responses to odors. Although these tools have been widely used in olfactory research (Porcherot et al. 2010; Baer et al. 2018; Hirama et al. 2025), to date, no studies have employed these scales to investigate affective dimensions of cross-modal visual-olfactory integration with images.

Despite the growing interest in this topic, previous research has typically focused on contrasts between pleasant and unpleasant odors in relation to visual cues. Functional magnetic resonance imaging studies have linked activity in the orbitofrontal cortex to subjective hedonic ratings of fragrance (Grabenhorst et al. 2007; Carlson et al. 2020). However, pleasant odors usually produce weaker effects in brain areas related to attention in contrast to unpleasant odors, which strongly engage brain networks involved in emotion, avoidance, and olfactory learning (Grabenhorst et al. 2007; Carlson et al. 2020). The effects of pleasant smells may also vary depending on contextual or individual differences in affective dimensions (Carlson et al. 2020), but the effects of such variance on hedonic ratings or evaluations are not fully understood. Moreover, framing odor perception as a binary pleasant-unpleasant contrast, as is common in many experiments, does not reflect complex naturalistic experiences, which are proposed to exist along a continuum (Amsellem et al. 2018).

The present study aimed to investigate whether pleasant odors, which vary in character, differentially influence the hedonic perception of visual stimuli, specifically faces, objects and landscapes, and whether the effect of these specific odors varies by visual categories. We also explored the individual dimensions of emotional responses evoked by these odors (e.g. nostalgia, sensuality) and how these might shape cross-modal interactions and evaluations of images. To date, no study has assessed how emotions induced by a range of pleasant odors interact with hedonic evaluations across multiple categories of images. To reduce potential variability associated with cross-cultural differences in olfactory associations, the study was conducted with participants based only in the United Kingdom.It was hypothesized that pleasant odors would increase pleasantness ratings for all image types, particularly for congruent positively valenced stimuli (Demattè et al. 2007; Cook et al. 2017; Syrjänen et al. 2021). Additionally, this study explored how the different affective dimensions of the LEOS scale account for the variability in hedonic evaluations of odor–visual stimuli, to explore specific dimensions of individual affective responses to odors as covariates for subsequent hedonic odor–visual evaluations in each of the visual stimuli categories.

## Methods

### Participants

A total of 40 participants (9 males) aged 18 to 35 yr (mean ± SD: 22.75 ± 4.33) took part in the current experiment after providing written informed consent in accordance with the Declaration of Helsinki. The study was approved by the University of Liverpool Health and Life Sciences Research Ethics Committee (ID: 12934). One participant was excluded from the analysis due to being identified as an outlier on self-report ratings of visual stimuli across all conditions (more than ±2.5 SD from mean), exhibiting a response pattern suggestive of systematic responding. This left a sample of 39.

Prior to the experiment, all participants underwent olfactory screening using the *Sniffin’ Sticks* test. Participants were asked to identify 12 test fragrances from a selection of 4 options for each. A minimum of 9 correct recognitions (out of 12 probes) were required to confirm normal sense of smell, which all participants achieved. Exclusion criteria included self-reported olfactory disorders (such as hyposmia or anosmia), and respiratory conditions (such as asthma), as well as a history of olfactory hallucinations or fragrance allergies. Participants were instructed to avoid smoking, eating, drinking, or chewing gum 2 h prior to the experiment, and to refrain from using fragranced products on the day of the experiment. Participants were reimbursed £13 for their time and travel expenses.

### Visual and olfactory stimuli

For face images, this experiment used 14 images of 7 actors (2 males, 5 females) from the Chicago Face Database (CFD; Ma et al. 2015), a standardized database of high-resolution photographs of faces with norming data (Singh et al. 2023). The database includes individuals of various genders and ethnicities showing different facial expressions (Ma et al. 2015). All actors wore gray t-shirts and their faces displayed neutral and happy (closed-mouth) expressions, with their head and shoulders squared to the camera. These expressions were validated using the database's norming data (Ma et al. 2015). All images were in color, sized at 1,086 × 724 pixels with luminance and contrast standardized against a white background. Although participant ethnicity was not measured or reported in this study, a diverse sample was anticipated, and image selection reflected this: 2 black actors and 5 white actors were selected. The final images were chosen through a pre-experiment screening session, with further details (including image codes) provided in Supplementary Appendix A.

In addition to faces, a total of 28 pictures of objects and landscapes (14 per category) were presented in this study. These were obtained from the International Affective Picture System (IAPS; Lang et al. 1993). The IAPS is an image dataset of landscapes, objects, scenes, and humans of different affective valence and intensity (Lang et al. 1993). All selected images were prototypical of either neutral (7 per category) or positive valence (7 per category) according to standardization data provided by the dataset (Lang et al. 2008). All pictures were unaltered in quality, measured 1,024 × 720 pixels, and excluded odor-related content (e.g. flowers, food) or inclusion of human presence to minimize bias effects. Landscape images were balanced between indoor and outdoor scenes. Supplementary Appendix B provides details on the image selection and the pre-experiment screening.

Odors were administered through 2 tubes approximately 2 cm away from the nostrils, using a custom-built, continuous airflow, computer-controlled olfactometer with 8 channels (Dancer Design Ltd., Ingleton, UK). Odor pulses were embedded within a constant flow of clean air to prevent airflow fluctuations during odor presentation (Huart et al. 2012). Airflow was maintained at a constant flow of 2.25 L/min, in line with previous studies from our laboratory (Cook et al. 2017; Davies-Owen et al. 2024).

This experiment included 5 odor conditions: 4 pleasant-valenced fragrances and a neutral “clean air” control. Vanilla (2% dilution in propylene glycol), rose (0.5%), lavender (0.2%), and lemon (0.2%) were selected for the pleasant conditions based on a scoping review which identified them as some of the most commonly pleasant odors in research (see Supplementary Appendix C). Furthermore, each fragrance represents a specific odor category—sweet, floral, aromatic, and citrus, respectively (Sharma et al. 2022). Fragrance dilutions were matched for perceived intensity using data from a prescreening session (see Supplementary Appendix D). Odors were supplied by Symrise Ltd. (Barneveld, the Netherlands) and propylene glycol (1,2-propanediol 99%, Sigma-Aldrich Ltd., Gillingham, Dorset, UK) was used as diluent. PsychoPy 2023.12.1 (RStudio Team, Boston, MA, USA) was employed to control image presentation and fragrance valves. To minimize residual odor exposure, a Blueair 203 air purifier (Blueair Ltd., Stockholm, Sweden) filtered the environment after each experimental task.

Participants rated each fragrance and control condition for pleasantness and intensity on 2 separate visual analog scales (VAS) ranging from 0 (*not at all*) to 10 (*very pleasant/intense*) with a midpoint of 5 (*mild/neutral*), while the odor was delivered via olfactometer. The questions and rating scales were programmed using PsychoPy 2023.12.1 (RStudio Team). The text was presented in white on a black background.

### Questionnaire

Participants completed a computerized version of the LEOS (Ferdenzi et al. 2011). This is a tool created to assess odor-induced emotions and adapted specifically for the UK population (Ferdenzi et al. 2013). The questionnaire evaluates 7 emotional domains relevant to a fragrance presented concurrently (happiness, disgust, nostalgia, thirstiness, relaxation, sensuality, and energy) using the question “In your opinion, is the term relevant to describing an emotional state you have already experienced when smelling this odour in the past?” Participants rated each fragrance and the control condition on a VAS ranging from 0 (*not at all*) to 10 (*very much*). If a question was unclear, they could select the “don’t understand” option, presented as a button. PsychoPy 2023.12.1 (RStudio Team) was used to present questions, record responses, and control odor valves. This interface displayed each question along with the corresponding VAS and the optional “don’t understand” button, simultaneously with the odor presentation, in line with recommendations of original authors. The text was presented in white on a black background. Moreover, each odor was delivered via the olfactometer and remained present until the participant submitted their rating for each dimension at the same flow rate used for the full experiment.

### Procedure

Participants were tested at the University of Liverpool between January and March 2024. Upon arrival at the Brain and Behaviour Laboratory, participants gave informed consent and were screened for allergies, asthma, and olfactory issues and then completed the olfactory screen using the Sniffin’ Sticks. Following a successful screening, participants were led to a dimly lit, sound-attenuated room and seated 0.7 m in front of a 19-inch LCD monitor (60 Hz refresh rate). The olfactometer headpiece was fitted, demographic data were acquired, and task instructions were provided.

**Figure 1:**
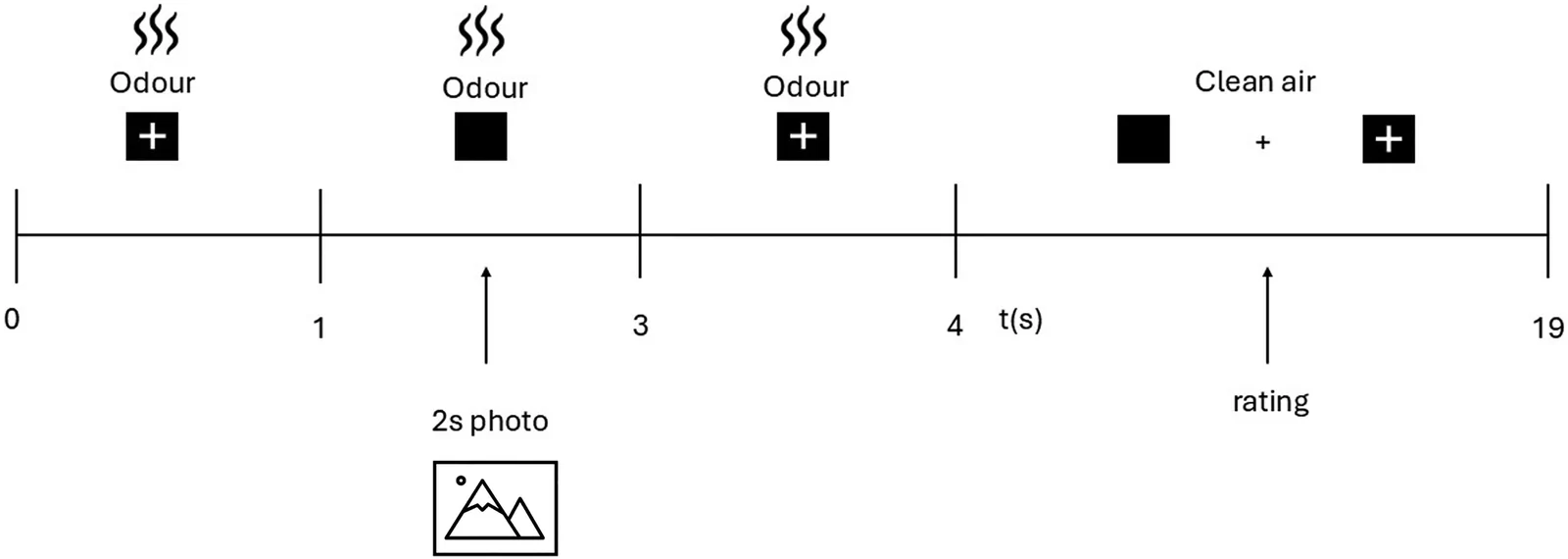
Flowchart of an experimental trial.

Each session lasted approximately 1 h 40 min and included baseline odor ratings, 5 experimental blocks (one per odor condition) and the LEOS questionnaires. Before each task, participants rated the pleasantness and intensity of the corresponding odor using an on-screen VAS from 0 (neutral) to 10 (very much), with a midpoint of 5 (mild/neutral). Each of the 5 tasks consisted of 42 trials (one per image), and they were fully randomized. Figure 1 provides a flowchart of the trial procedure. Each trial began with a 20 s resting interval, displaying a white cross on a black background. An odor pulse was then administered for 4 s. During this time, participants first viewed a white fixation cross on a black background (1 s), then one of the images (2 s), followed by another white fixation cross (1 s). Immediately after, they rated the image's pleasantness on a screen VAS from 0 (*neutral*) to 10 (*very pleasant*) using a mouse, then a final resting period followed. The length of this final interval was adapted depending on how long participants took to rate, to ensure that a minimum interstimulus interval of 15 s while clean air was delivered. Image ratings were collected during this clean-air interval immediately after stimuli delivery to minimize potential cognitive and perceptual confounds associated to simultaneous odor exposure, image visualization and behavioral responding (Forster and Spence 2018). This timing structure was also intended to ensure that responses were based on image evaluation rather than the immediate experience of odor (Oleszkiewicz et al. 2018). Importantly, this does not prevent any odor-related modulation, as olfactory influences on visual evaluations can persist across short stimulus-onset asynchronies, with evidence showing these odor effects on visual evaluation lasting even after the physical stimulus is no longer present (Cook et al. 2018). Moreover, this clean-air interval was included to reduce the risk of olfactory desensitization, which could affect the processing of subsequent images (Fallon et al. 2020). Immediately after each block, participants completed the LEOS questionnaire for the corresponding odor, using the provided mouse. A 1-min pause occurred between the task block and the questionnaire to allow time for explaining the new instructions, during which clean air was delivered to maintain the airflow.

### Analysis

All analyses were conducted in RStudio 2023.12.1 (RStudio Team) with the lmerTest package (Kuznetsova et al. 2017) and the lme4 package (Bates et al. 2015). Moreover, all significant thresholds were set at *P* <0.05. Ratings of odor pleasantness and intensity taken prior to the experimental tasks were analyzed using 1-way repeated-measures analysis of variance (ANOVA).

Image ratings were analyzed using linear mixed-effect models, with repeated observations nested within participants and stimuli (image trials). Fixed effects included odor conditions (vanilla, rose, lavender, lemon, and clean air), image type (faces, landscapes, and objects), image valence (neutral and positive), and their interactions. Random-slope structures were tested but removed due to singular fits and convergence issues. Model comparison showed that the mixed-effects model provided substantially better fit than the fixed-effects-only model (*ΔAIC* = 578.86). The final model is specified as:


$$Rating_{ijk}=\beta_{0}+\beta_{1}\cdot Odour_{i}+\beta_{2\;}\cdot Valence_{i}+\beta_{3}\cdot Type+\beta_{4}\cdot (Odour\cdot Valence\cdot Type{)}_{i}+u_{j}+v_{k}+e_{ijk}$$

where: β_1,2,3,4_ = fixed effects coefficients, *i* = observations, *j* = participants, *k* = images, *u_j_* = random intercepts for participants, *v_k_* = random intercepts for images, *e_ijk_* = residual error.

Satterthwaite's method was used to estimate degrees of freedom as a default function of the lmer package. Significant interaction effects were explored using post-hoc comparisons.

To better understand the role of odor-evoked emotional associations, the mixed-effects modeling framework was extended to include the 7 LEOS questionnaire items as covariates of interest to investigate whether subjective emotional profiles influenced pleasantness ratings across odor conditions and image categories. The LEOS covariates were entered as fixed effects because a correlation matrix (found in Supplementary Appendix G) indicated no evidence of multicollinearity (all Pearson's *r* < .70; Dormann et al. 2013). The fixed effects included odor condition (vanilla, rose, lavender, lemon, and clean air), image valence (neutral, positive), and image type (faces, landscapes, and objects), and all interactions among experimental factors. Random intercepts were included for both participants and image trials. The following formula was applied using the Type III ANOVA function in RStudio 2023.12.1 (RStudio Team) with the lmerTest package (Kuznetsova et al. 2017):


$$\begin{array}{rl} & Rating_{ijk}=\beta_{0}+\beta_{1}\cdot Odour_{i}+\beta_{2\;}\cdot Valence_{i}\;+\beta_{3}\cdot Type\\ & \;+\beta_{4}\cdot (Odour\cdot Valence\cdot Type{)}_{i}+\;\gamma_{1\;}\cdot Happiness_{j}\\ & \;+\gamma_{2\;}\cdot Disgust_{j}+\gamma_{3\;}\cdot Nostalgia_{j}\;+\;\gamma_{4\;}\cdot Thirstiness_{j}\\ & \;+\;\gamma_{5}\cdot Relax_{j}+\;\gamma_{6}\cdot Sensuality_{j}+\;\gamma_{7\;}\cdot Energy_{j}+u_{j}+v_{k}+e_{ijk} \end{array}$$

Where: β_1,2,3,4_ = fixed effects coefficients for odor, valence, type, $\gamma_{1-7}$ = fixed effects coefficient for the 7 LEOS covariates, *i* = observations, *j* = participants, *k* = images, *u_j_* = random intercepts for participants, *v_k_* = random intercepts for images, *e_ijk_* = residual error.

Finally, LEOS questionnaire ratings were analyzed using a series of 2-way repeated-measures ANOVA to explore how emotional response (happiness, disgust, nostalgia, thirstiness, relaxation, sensuality, and energy) differed across odors. Significant interaction effects were explored using post-hoc comparisons.

## Results

### Baseline odor ratings

Descriptive statistics for the pleasantness and intensity ratings (VAS 0 to 10) of each odor are presented in Table 1.

**Table 1:** Pleasantness and intensity descriptive statistics and ANOVA summaries across odors.

Odor	Pleasantness mean (SD)	Intensity mean (SD)
Clean air	5.13 (1.55)	1.99 (2.29)
Vanilla	7.00 (2.56)	5.90 (2.41)
Rose	6.42 (2.28)	5.21 (2.02)
Lavender	6.36 (2.53)	5.12 (2.24)
Lemon	5.64 (2.39)	5.55 (2.20)

**Figure 2:**
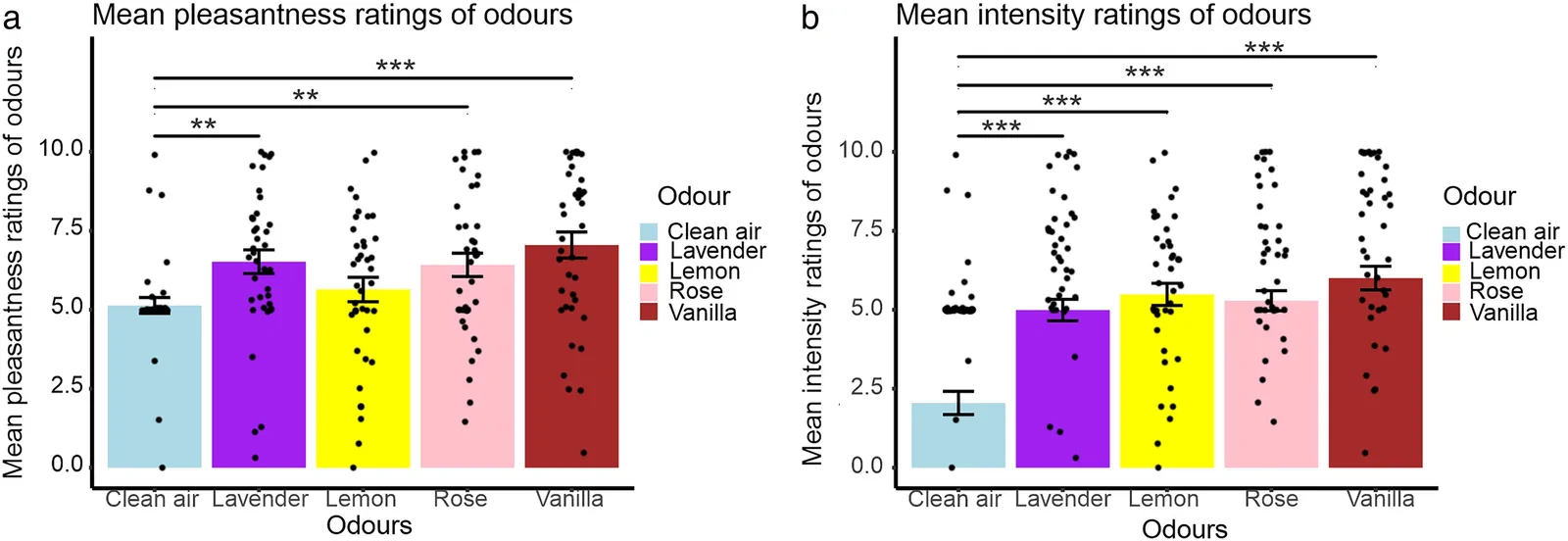
Bar plots for descriptive statistics of pleasantness and intensity for each odor. These bar plots show means and standard deviations for a) pleasantness ratings and b) intensity ratings for each odor. Asterisks indicate significant difference between odors and clean air (control) (post-hoc pairwise *t*-tests), with *P < 0.05, **P < 0.01, and ***P < 0.001.

A 1-way repeated-measures ANOVA revealed a significant effect of odor type on pleasantness ratings (*F*(4, 152) = 4.37, *P* = 0.002, $\eta_{p}^{2}$ = 0.08). Post-hoc comparisons suggest that lavender (*t*(76) = 3.07, *P* = 0.003, Cohen's *d* = 0.69, 95% CI [0.22, 1.16]) rose (*t*(76) = 2.87, *P* = 0.005, Cohen's *d* = 0.65, 95% CI [0.16, 1.14]), and vanilla (*t*(76) = 3.96, *P* < 0.001, Cohen's *d* = 0.91, 95% CI [0.32, 1.50]) were rated significantly more pleasant than clean air, whereas lemon (*t*(76) = 1.09, *P* = 0.277, Cohen's *d* = 0.25, 95% CI [−0.24, 0.74]) did not differ in terms of pleasantness from the clean air condition. The bar chart in Fig. 2a illustrates differences in pleasantness ratings for each odor. A 1-way repeated-measures ANOVA also showed a significant effect of odor on intensity (*F*(4, 152) = 27.00, *P* < 0.001, $\eta_{p}^{2}$ = 0.29). Post-hoc comparisons suggest that all the fragrances were rated significantly more intense than clean air: lavender *t*(76) = 5.87, *P* < 0.001, Cohen's *d* = 1.33, 95% CI [0.70, 1.96]; rose *t*(76) = 6.67, *P* < 0.001, Cohen's *d* = 1.51, 95% CI [0.88, 2.13]; vanilla *t*(76) = 7.52, *P* < 0.001, Cohen's *d* = 1.70, 95% CI [1.03, 2.38]; and lemon *t*(76) = 6.77, *P* < 0.001, Cohen's *d* = 1.53, 95% CI [0.86, 2.21]. These results are illustrated in the bar chart in Fig. 2b.

### Image ratings during fragrance presentation

**Figure 3:**
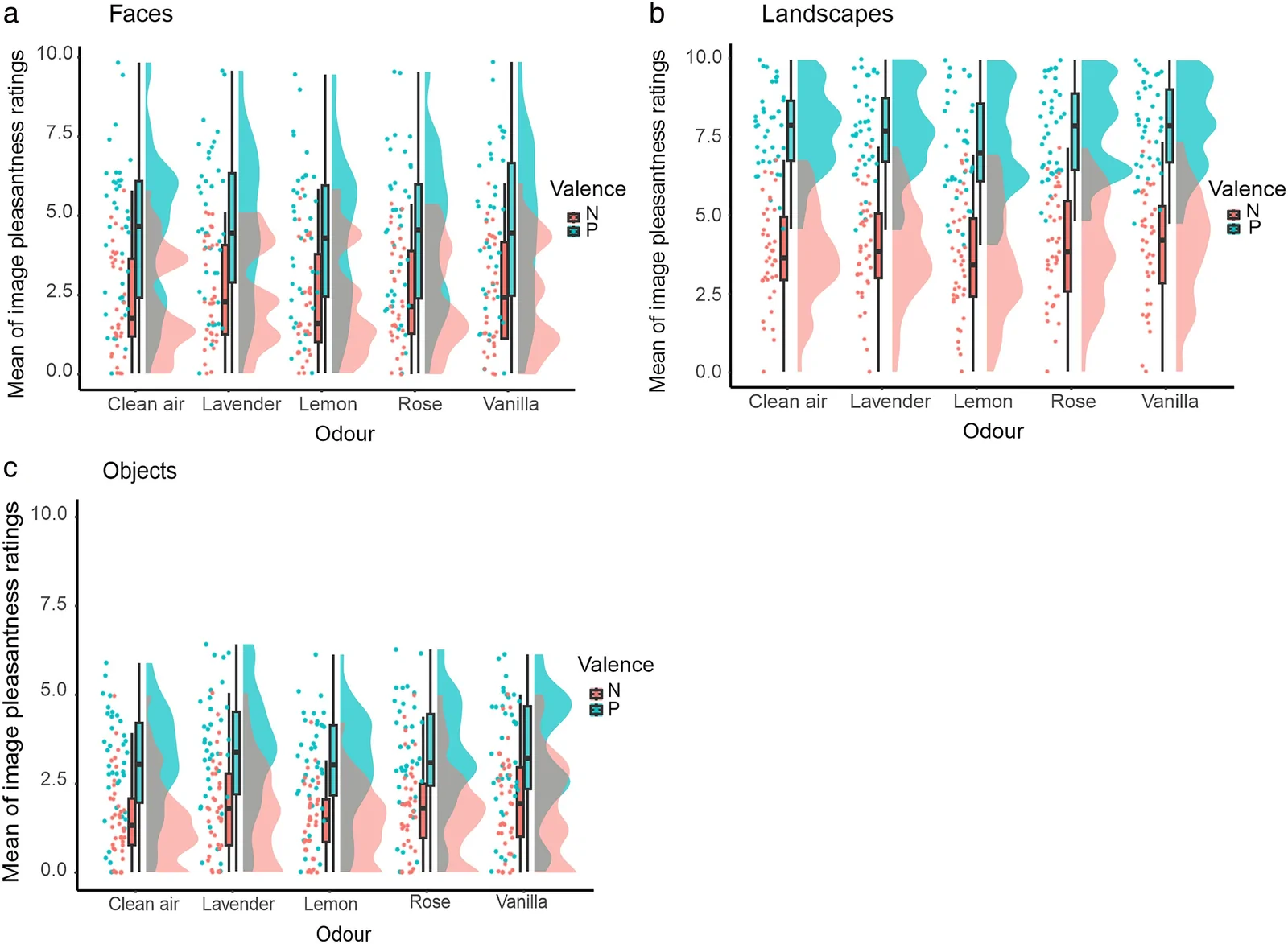
Raincloud plot of image pleasantness ratings for each category of visual image. N = Neutral, P = Positive. This raincloud plot shows the distribution of mean subjective ratings for each odor, and valence (positive/neutral) of a) faces, b) landscapes, and c) objects. The half violin plots show the probability distribution of data, while the individual dots show mean data points for each participant. The box plot indicates the median and interquartile range (IQR) between the 25th and 75th percentile.

Descriptive statistics for image pleasantness ratings by type (faces, landscapes, objects), odor, and valence (neutral, positive) are provided in Supplementary Appendix E. Figure 3 presents raincloud plots illustrating the distribution of the mean subjective pleasantness ratings for each image category by odor and valence. Individual dots represent mean pleasantness ratings per participant in each odor and picture valence condition.

The linear mixed-effect model revealed a main effect of image valence (*F*(1, 100) = 118.68, *P* < 0.001, $\eta_{p}^{2}$ = 0.54), with positive images rated as more pleasant than neutral images, as shown in Fig. 3. Moreover, there were significant main effects of odor (*F*(4, 8,252.7) = 11.69, *P* < 0.001, $\eta_{p}^{2}$ < 0.01) and image type (*F*(2, 94) = 139.13, *P* < 0.001, $\eta_{p}^{2}$ = 0.75). The interaction between odor and image type was also significant (*F*(8, 8,252.7) = 2.59, *P* = 0.008, $\eta_{p}^{2}$ < 0.01), suggesting that odor effects varied across faces, landscapes, and objects. Exploratory follow-up pairwise comparisons showed that faces were rated significantly more pleasant in the presence of lavender compared to the clean air condition (*t*(8,253) = 2.05, *P* = 0.040, Cohen's *d* = 0.12, 95% CI [0.01, 0.50]). Moreover, faces were rated significantly more pleasant in the presence of lavender (*t*(8,253) = 3.09, *P* = 0.002, Cohen's *d* = 0.18, 95% CI [0.14, 0.63]), and vanilla (*t*(8,253) = 2.67, *P* = 0.008, Cohen's *d* = 0.16, 95% CI [0.09, 0.58]) compared to the lemon odor condition.

Furthermore, the results showed that landscapes were rated significantly more pleasant during lavender (*t*(8,253) = 3.24, *P* = 0.001, Cohen's *d* = 0.19, 95% CI [0.16, 0.65]), rose (*t*(8,253) = 4.85, *P* < 0.001, Cohen's *d* = 0.30, 95% CI [0.36, 0.85]), and vanilla (*t*(8,253) = 5.39, *P* < 0.001, Cohen's *d* = 0.32, 95% CI [0.43, 0.92]) compared to lemon. Notably, landscape pictures were rated as less pleasant in the presence of lemon compared to clean air (*t*(8,253) = −4.51, *P* < 0.001, Cohen's *d* = 0.27, 95% CI [−0.81, −0.32]). No other odor differed from the clean air condition (*P* > 0.05). Moreover, landscapes were rated significantly more pleasant during the vanilla condition compared to lavender (*t*(8,253) = 2.14, *P* = 0.032, Cohen's *d* = 0.13, 95% CI [0.02, 0.51]).

The pairwise comparisons also showed that objects were rated significantly more pleasant during lavender (*t*(8,253) = 2.37, *P* = 0.018, Cohen's *d* = 0.14, 95% CI [0.05, 0.54]), rose (*t*(8,253) = 2.69, *P* = 0.007, Cohen's *d* = 0.16, 95% CI [0.09, 0.58]), and vanilla (*t*(8,253) = 3.44, *P* < 0.001, Cohen's *d* = 0.21, 95% CI [0.18, 0.67]) compared to clean air. Moreover, objects were rated significantly more pleasant in the presence of rose (*t*(8,253) = 2.17, *P* = 0.030, Cohen's *d* = 0.13, 95% CI [0.03, 0.52]) and vanilla (*t*(8,253) = 2.91, *P* = 0.004, Cohen's *d* = 0.17, 95% CI [0.12, 0.61]) compared to lemon.

Additionally, the interaction between image valence and image type was significant (*F*(2,100) = 37.60, *P* < 0.001, $\eta_{p}^{2}$ = 0.36), indicating that the effect of valence differed across image categories. Exploratory follow-up pairwise comparisons showed that positive pictures were rated significantly higher than neutral pictures in landscapes (*t*(103.3) = 12.38, *P* < 0.001, Cohen's *d* = 2.12, 95% CI [3.73, 5.15]), in faces (*t*(98.3) = 3.97, *P* = 0.001, Cohen's *d* = 0.70, 95% CI [0.72, 2.16]), and in objects (*t*(98.3) = 2.60, *P* = 0.011, Cohen's *d* = 0.45, 95% CI [0.22, 1.66]).

In contrast, the interaction between odor and image valence (*F*(4,8,252.7) = 1.53, *P* = 0.191, $\eta_{p}^{2}$ < 0.01), and the 3-way interaction among odor, image valence and image type (*F*(8,8,252.7) = 0.28, *P* = 0.971, $\eta_{p}^{2}$ < 0.01) was not significant. Full model estimates for fixed and random effects are reported in Supplementary Appendix H.

### Emotional associations with image ratings during fragrance presentation

After including all LEOS emotional dimensions (happiness, disgust, nostalgia, thirstiness, relaxation, sensuality, and energy) as covariates in the model, the main effect of image valence on pleasantness ratings remained significant (*F*(1, 91.99) = 124.96, *P* < 0.001, $\eta_{p}^{2}$ = 0.58), and the main effect of image type was also significant (*F*(2, 86.69) = 145.55, *P* < 0.001, $\eta_{p}^{2}$ = 0.77). However, the main effect of odor remained significant, although it was substantially reduced (*F*(4, 8,213.26) = 2.56, *P* = 0.037, $\eta_{p}^{2}$ < 0.01). This indicates that the variance previously attributed to odor overlapped with participants’ emotional responses to the fragrances, such that controlling for LEOS dimensions demonstrates a reduction of this shared variance rather than indicating a causal mechanism of odor effects.

Moreover, subjective levels of happiness (*F*(1, 8,256.01) = 12.52, *P* < 0.001, $\eta_{p}^{2}$ < 0.01), nostalgia (*F*(1, 8,287.69) = 28.12, *P* < 0.001, $\eta_{p}^{2}$ < 0.01), disgust (*F*(1, 6,572.66) = 73.56, *P* < 0.001, $\eta_{p}^{2}$ = 0.01), and thirstiness (*F*(1, 7,365.11) = 7.49, *P* = 0.006, $\eta_{p}^{2}$ < 0.01) ratings were significantly associated with variance in image pleasantness ratings that covaried with odor-related differences. However, relaxation (*F*(1, 8,163.05) = 3.76, *P* = 0.052, $\eta_{p}^{2}$ < 0.01), sensuality (*F*(1, 7,534.10) = 2.15, *P* = 0.143, $\eta_{p}^{2}$ < 0.01), and energy (*F*(1, 7,926.86) = 0.98, *P* = 0.322, $\eta_{p}^{2}$ < 0.01) ratings did not account for a significant portion of variance in image pleasantness associated with odor-related differences.

Finally, the interaction between odor and image type was significant (*F*(8, 8,269.35) = 2.67, *P* = 0.006, $\eta_{p}^{2}$ < 0.01), suggesting that odor effects varied across image categories. Exploratory follow-up pairwise comparisons showed that, after accounting for the LEOS covariates in the model, faces were rated significantly more pleasant in the presence of lavender compared to the rose condition (*t*(8,276) = 2.02, *P* = 0.043, Cohen's *d* = 0.12, 95% CI [0.02, 0.24]). Moreover, landscapes were rated significantly more pleasant in the presence of rose (*t*(8,292) = 3.36, *P* = 0.008, Cohen's *d* = 0.21, 95% CI [0.09, 0.33]), and vanilla (*t*(8,292) = 3.57, *P* = 0.004, Cohen's *d* = 0.22, 95% CI [0.10, 0.35]) compared to the lemon odor condition. In addition, landscapes were significantly less pleasant in the presence of lemon (*t*(8,304) = −4.13, *P* < 0.001, Cohen's *d* = −0.27, 95% CI [−0.39, −0.14]) compared to clean air, and significantly more pleasant in the presence of lavender (*t*(8,310) = 2.31, *P* = 0.021, Cohen's *d* = 0.15, 95% CI [0.02, 0.27]) compared to clean air. Furthermore, none of the odor pairs differed significantly for object images (all *P* > 0.05).

**Figure 4:**
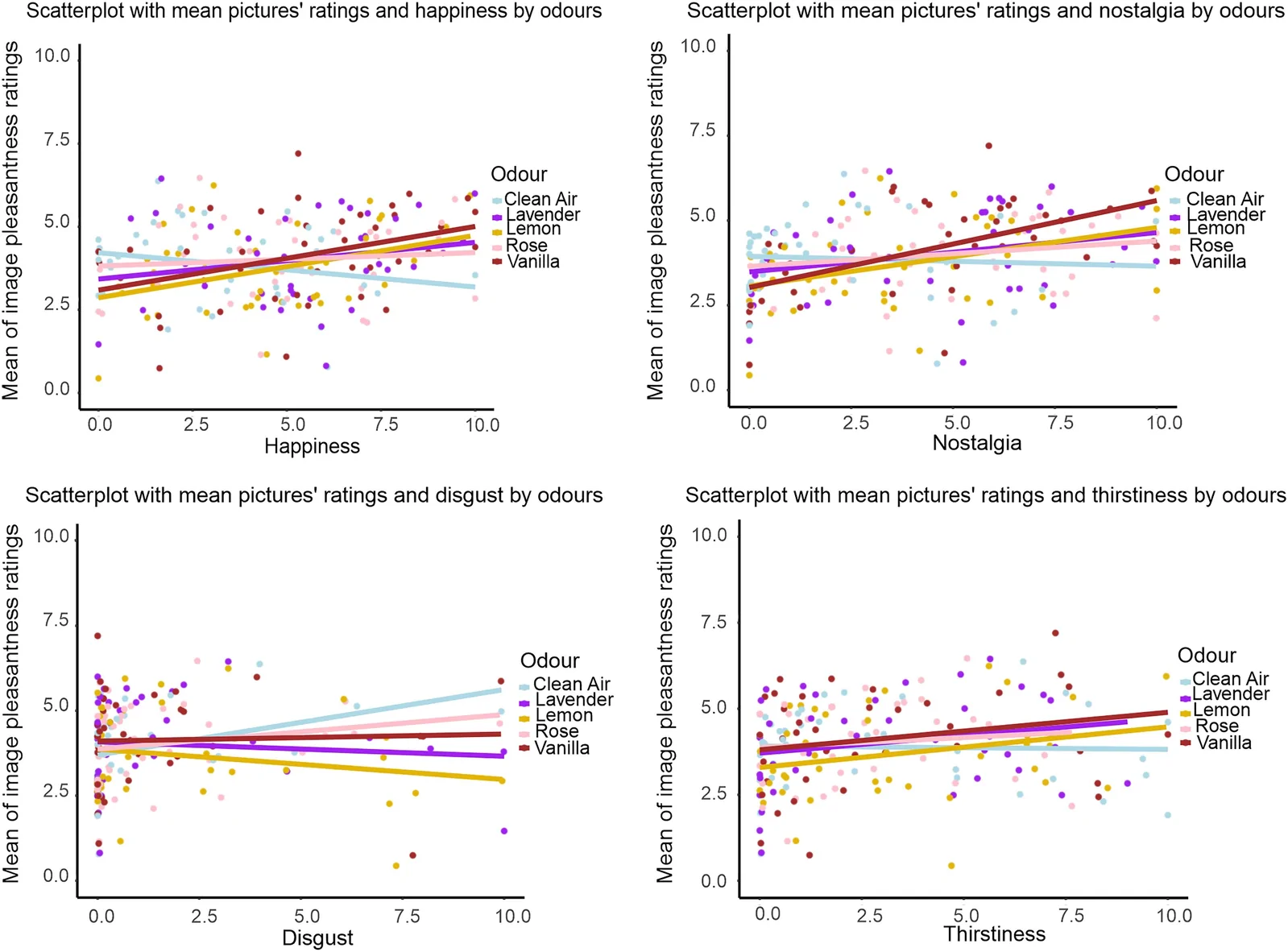
Scatterplots of covariates by odors for image ratings. Scatterplots showing how LEOS ratings of affective responses to odors relate to mean pleasantness ratings. Affective dimensions demonstrating a significant effect on ratings are shown for each category.

The interaction between image valence and image type was also significant (*F*(2, 91.99) = 28.15, *P* < 0.001, $\eta_{p}^{2}$ = 0.38), indicating that the effect of valence differed across image categories. Exploratory follow-up pairwise comparison showed that, after accounting for the LEOS covariates in the model, positive pictures were rated significantly higher than neutral in landscapes (*t*(94.9) = 12.59, *P* < 0.001, Cohen's *d* = 2.13, 95% CI [1.79, 2.46]), in faces (*t*(90.6) = 4.15, *P* < 0.001, Cohen's *d* = 0.71, 95% CI [0.37, 1.05]), and in objects (*t*(90.6) = 2.69, *P* = 0.034, Cohen's *d* = 0.46, 95% CI [0.12, 0.80]). The interaction between odor and image valence was not significant (*F*(4, 2,416.41) = 0.99, *P* = 0.412, $\eta_{p}^{2}$ < 0.01), and the 3-way interaction among odor, image valence and image type was also not significant (*F*(8, 8,269.36) = 0.29, *P* = 0.970, $\eta_{p}^{2}$ < 0.01). Complete analysis of covariance (ANCOVA) model results are found in Table 2. Moreover, Fig. 4 presents scatterplots of the significant LEOS covariates for the image ratings, showing a positive association between happiness, nostalgia, and thirstiness ratings and the effects of odors on images. Furthermore, it shows a negative association between disgust ratings and odor effects on images, particularly for the lemon condition, compared to clean air. Finally, full model estimates for fixed and random effects of the ANCOVA model are reported in Supplementary Appendix H.

**Table 2:** Mixed-effects ANCOVA model.

Variable/covariate	*df_num_*	*df_den_*	*F*	*P*
Odor	4	8,213.26	2.56	0.037
Valence	1	91.99	124.96	<0.001*
Type	2	86.69	145.55	<0.001*
Happiness	1	8,256.01	12.524	<0.001*
Nostalgia	1	8,287.69	28.12	<0.001*
Disgust	1	6,572.66	73.56	<0.001*
Relaxation	1	8,163.05	3.76	0.052
Sensuality	1	7,534.11	2.15	0.142
Thirstiness	1	7,365.11	7.49	0.006*
Energy	1	7,926.86	0.98	0.322
Odour:Valence	4	8,269.36	1.57	0.178
Odour:Type	8	8,269.36	2.67	0.006*
Valence:Type	2	92.00	28.15	<0.001*
Odour:Valence:Type	8	8,269.36	0.29	0.970

Analysis conducted with Satterthwaite's method. * = significant.

### Questionnaire ratings during fragrance presentation

**Figure 5:**
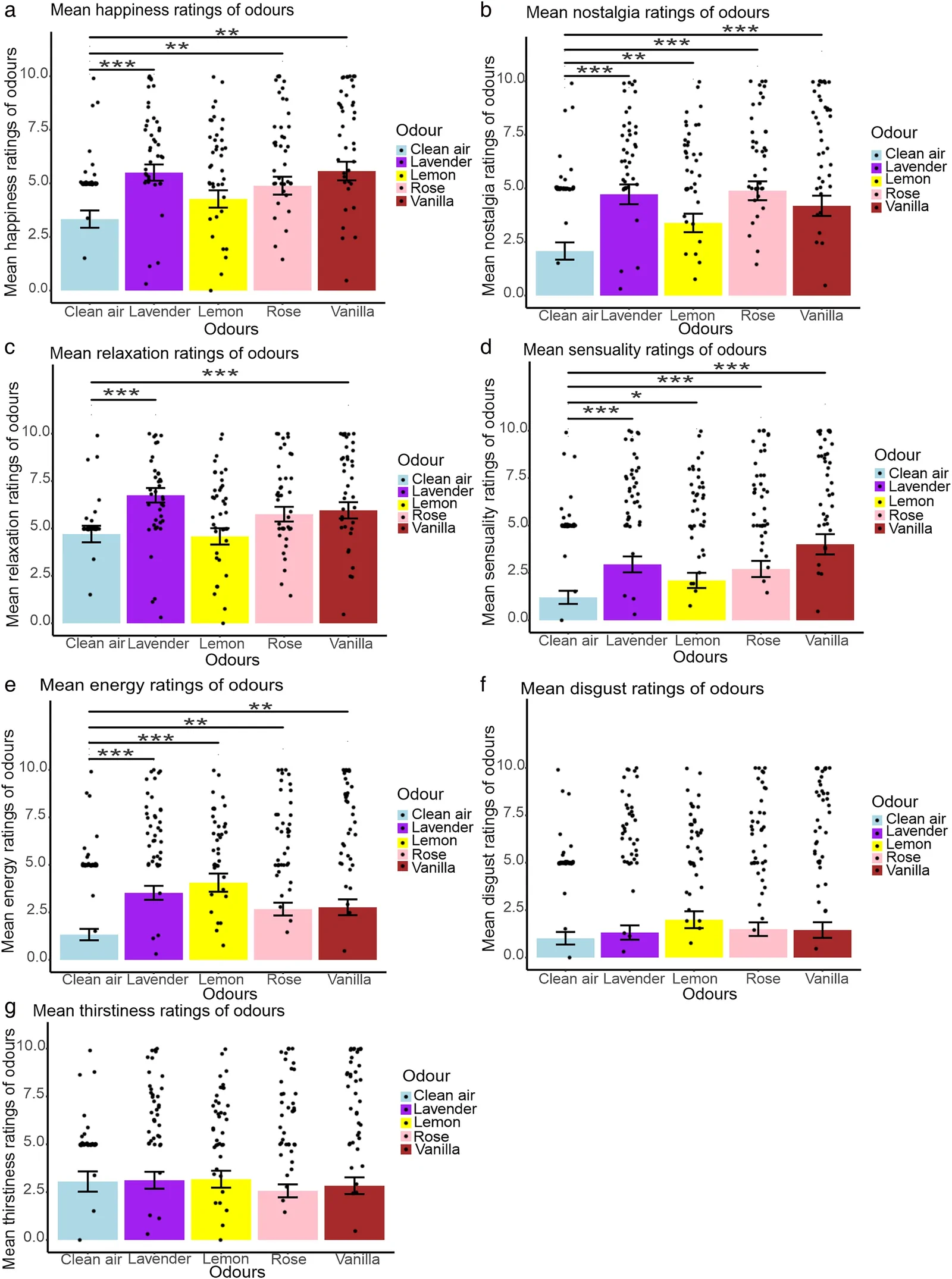
Bar plots showing means and standard deviations of LEOS's affective domains ratings across odors. Panels show a) happiness, b) nostalgia, c) relaxation, d) sensuality, e) energy, f) disgust, g) thirstiness for each odor. Asterisks indicate significant difference between odors and clean air (control) (post-hoc pairwise *t*-tests), with *P < 0.05, **P < 0.01, and ***P < 0.001.

Descriptive statistics for each LEOS emotional domain by odor are provided in Supplementary Appendix F. A series of 1-way ANOVAs (using the multilevel model with Satterthwaite's method) were conducted to assess the main effects of odor on each emotional dimension rating. Significant effects of odor were found for happiness (*F*(4, 152) = 6.97, *P* < 0.001, $\eta_{p}^{2}$ = 0.10), nostalgia (*F*(4, 152) = 8.98, *P* < 0.001, $\eta_{p}^{2}$ = 0.12), relaxation (*F*(4, 152) = 6.23, *P* < 0.001, $\eta_{p}^{2}$ = 0.09), sensuality (*F*(4, 152) = 12.14, *P* < 0.001, $\eta_{p}^{2}$ = 0.11), energy (*F*(4, 152) = 11.87, *P* < 0.001, $\eta_{p}^{2}$ = 0.13). However, no significant effects were observed for disgust (*F*(4, 152) = 1.75, *P* = 0.141, $\eta_{p}^{2}$ = 0.02) or thirstiness (*F*(4, 152) = 0.86, *P* = 0.488, $\eta_{p}^{2}$ = 0.01). This is shown in Fig. 5. Post-hoc pairwise exploratory comparisons for each LEOS dimension rating are provided in Supplementary Appendix I.

## Discussion

The present study examined whether pleasant odors, which vary in character, differentially influence hedonic evaluations of 3 categories of positively and neutrally valenced visual stimuli. We also investigated the role of individual differences in odor-specific emotional dimensions, and how these related to the impact of odors on evaluations. The study design employed a range of pleasant fragrances and clean air control and found that odors affected evaluation in a nuanced way that varied for different categories of images. Images were often rated as more pleasant in the presence of fragrances associated with more positive affective appraisal compared to the control (clean air) and lemon, which showed lower ratings in both baseline pleasantness and LEOS dimensions in the current sample. However, the different categories of images produced different results, suggesting that contextual factors influence the role of specific fragrance in cross-modal evaluations of visual scenes in addition to valence.

Specifically, faces were rated as more pleasant in the presence of lavender compared to clean air, and they were also rated as more pleasant in the presence of lavender and vanilla compared to lemon. Landscapes were not rated as more pleasant in any fragrance condition compared to the control, but they were rated as more pleasant in the presence of lavender, rose and vanilla compared to lemon. Moreover, landscapes were rated as more pleasant in the presence of vanilla compared to lavender. Finally, objects received higher hedonic ratings during the lavender, rose, and vanilla condition compared to clean air, and were also perceived as more pleasant during the presence of rose and vanilla compared to lemon.

The present study extends previous research on subjective affective responses to odor by examining how specific emotional dimensions, modeled as covariates, might relate to hedonic ratings of pictures. We found that several fragrance-evoked emotional dimensions included as covariates (happiness, nostalgia, disgust, and thirstiness) were significantly associated with variation in image pleasantness ratings. Indeed, the pattern of results suggests that odor-related differences in pleasantness image ratings shared variance with participants’ emotional responses to fragrances. However, the interaction between odor and image type remained significant, suggesting that these fragrance-related differences in pleasantness ratings varied across image categories even after accounting for the LEOS affective dimensions. Indeed, including these dimensions as covariates reduced the previously observed odor effects on pleasantness ratings for images, suggesting that odor-related differences overlapped with participants’ affective responses to the fragrances. This suggests that individual levels of affective dimensions evoked by a fragrance are critically important when it comes to understanding how odors influence hedonic judgments of different visual scenes.

### Effects of odors on the evaluation of the pictures

As hypothesized, odors impacted image pleasantness ratings; however, this effect differed by image category. For faces, ratings were higher when paired with lavender or vanilla than with lemon, and only lavender increased ratings compared to the control (clean air) condition. For landscapes, pictures were rated higher in the presence of vanilla, rose, or lavender than with lemon, with no difference observed compared to the clean air control. Moreover, landscapes were rated higher during vanilla, compared to lavender. In contrast, for objects, images were rated higher during the exposure to lavender, rose, or vanilla compared to clean air, with rose and vanilla also demonstrating increased ratings compared to lemon. These results suggest that hedonic evaluations of faces and objects were boosted in the presence of fragrances associated with more positive subjective appraisal compared to clean air and compared to lemon, whereas effects for landscapes appear to be mainly due to lemon's distinctive affective profile found in this sample, which exhibited lower ratings compared to the other odors and the control, rather than a systematic increase in pleasantness associated with the other fragrances. These differences may also reflect how each category is evaluated. In the present study, objects show broad improvements in pleasantness when paired with positively appraised odors; consistent with evidence that object perception relies heavily on contextual cues (Bar 2004; Lebrecht et al. 2012). However, faces carry social meaning and may only be more selectively boosted by certain fragrance qualities (such as odor-evoked emotional associations) rather than overall pleasantness alone. This is because faces are processed by specialized neural systems, meaning that their evaluations may be less susceptible to broad contextual influences than other visual stimuli (Kanwisher et al. 1997; Todorov et al. 2008). Landscapes, on the other hand, tend to elicit consistently positive affect (Ulrich 1983; Valtchanov et al. 2010), meaning that odor effects may emerge preferentially when a fragrance was perceived as less pleasant than others.

These findings are partly in line with previous literature (Demattè et al. 2007; Cook et al. 2015; Chen and Spence 2022), which showed that pairing faces with a pleasant odor resulted in enhanced attractiveness due to valence congruency. Lavender was the only fragrance to outperform clean air. However, no studies to date have specifically investigated how lavender influences hedonic evaluations of facial expressions. One interesting study reported that hexanal, a component shared by lavender smell and body odors, increased the perceived trustworthiness of facial expressions, which might be related to hedonic perception (Nieuwenburg et al. 2019). Lavender, rose and vanilla all demonstrated increased hedonic evaluations of faces compared to lemon odor. This may be due to how lemon was perceived in our sample, as it was not consistently considered pleasant in behavioral ratings. However, these findings may reflect differences in subjective affective profiles rather than fragrance identity alone, as further explored in relation to the emotional dimensions induced by the odors. Interestingly, previous studies have shown that lemon is associated with pleasantness, and also enhances the perceived pleasantness of happy faces (Winston et al. 2005; Li et al. 2020; Mancini et al. 2021). However, most of these studies have investigated the hedonic properties of lemon in comparison to unpleasant odors, which might have altered the nature of the impact of the fragrance on evaluations (Winston et al. 2005; Li et al. 2020). On the other hand, Mancini et al. (2021) compared lemon with lavender (another pleasant fragrance), and reported no differences between fragrance effects on evaluations of the pleasantness of VR experiences, although in this study additional factors such as sounds and movements may have influenced the odor's effects. Our results for landscapes and objects are broadly in line with previous studies (Walla and Deecke 2010; Adolph and Pause 2012; Jiang et al. 2016), although the literature regarding these image categories is still limited. To further clarify if the odor effects observed in the main analysis reflected meaningful differences among pleasant odors, complementary analyses were conducted excluding the lemon condition, and these are reported in Supplementary Appendix J. The results suggest that, once lemon was removed, odor-related differences generally became smaller, indicating that some of the variance was driven by the presence of lemon. However, some pairwise differences among lavender, vanilla, and rose remained, suggesting that these pleasant odors still subtly differ in how they influence hedonic evaluations.

Moreover, pleasant odors did not differentially impact ratings for positive images than neutral images across all 3 image categories. This might relate to a lack of observed congruency between olfactory and visual stimuli. Odor–visual congruence has been shown to have an important impact on perception of odor, as demonstrated by Fallon et al. (2020) and may also influence the evaluation of multisensory stimuli, although evidence for congruency effects is mixed (Syrjänen et al. 2021). One possible explanation for the current findings is that the present study did not include strongly congruent or incongruent odor–visual pairings (as this was controlled during image selection) reducing the likelihood of observing strong congruency-dependent effects.

### Affective dimensions induced by pleasant odors

**Figure 6:**
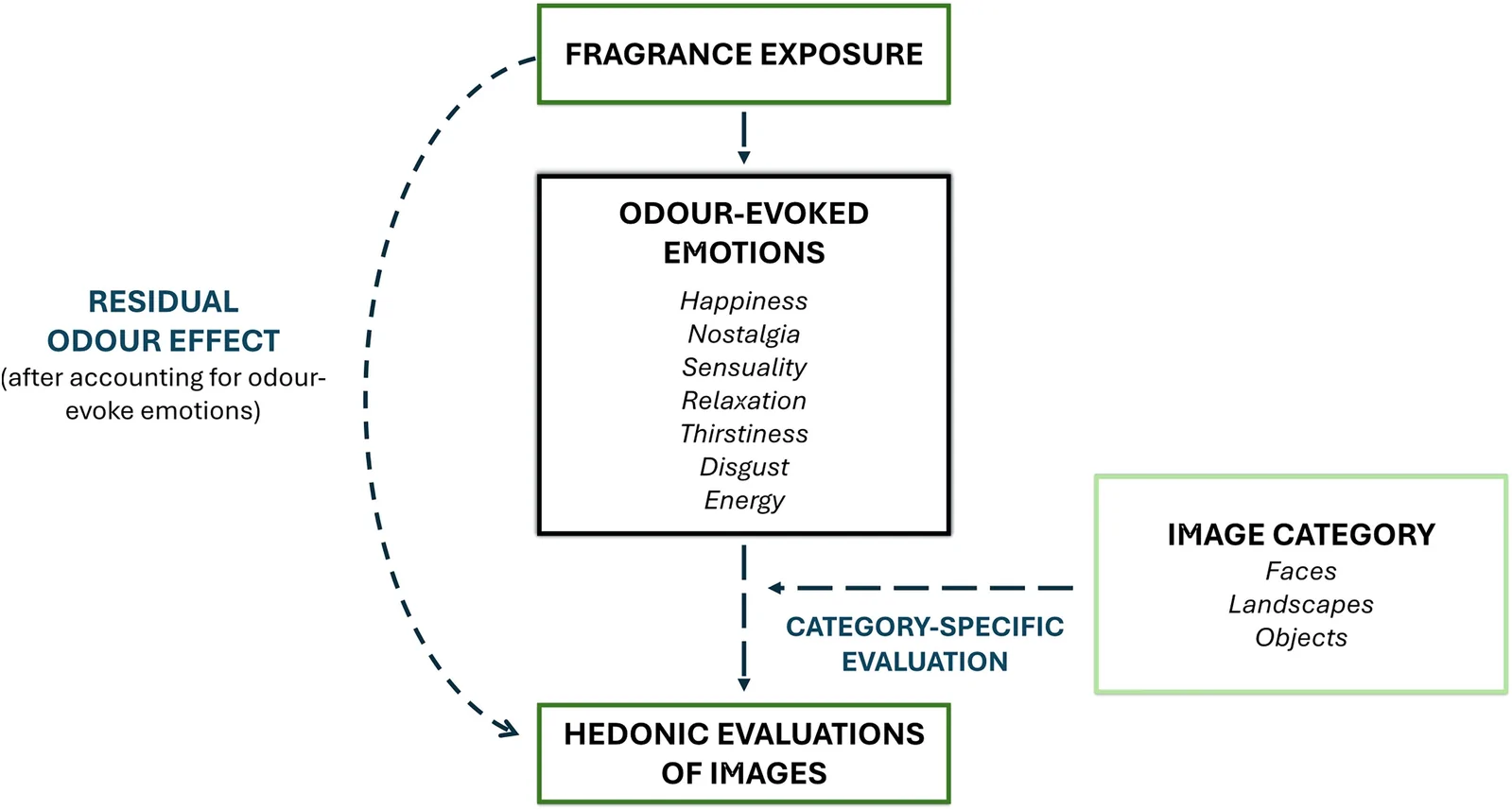
Schematic model illustrating the proposed mechanisms through which odors influence hedonic evaluations of images. Specifically, odor may affect evaluations indirectly through odor-evoked emotions, as well as through residual odor-related influences not captured by the emotional dimensions measured in the present study. These relationships also vary with image category.

Another novelty of the present research was the aim to integrate emotional dimensions associated with odor perception (happiness, nostalgia, disgust, relaxation, sensuality, thirstiness, and energy) as covariates into the analysis of how fragrance influences perceived pleasantness of images. Interestingly, when these variables were included, the main effects of odor remained significant but were substantially reduced, suggesting that individual variability in these 7 emotional domains is a key factor in the impact of odor on evaluations of images. Similar emphasis on individual differences in fragrance responses has been reported previously, suggesting that subjective responses to odors may be more important than general fragrance character (Sabiniewicz et al. 2022). Figure 6 shows a schematic model summarizing proposed mechanisms for the role of subjective affective associations for odor effects on hedonic evaluations of visual stimuli.

However, after accounting for the LEOS covariates in the model, odor effects varied across image categories, indicating that the influence of fragrances on visual evaluations is not uniform across visual types. Previous studies have shown specifically how faces can be particularly sensitive to odor-evoked affective states: for instance, Seubert et al. (2014) found that odors can modulate attractiveness of faces; and Davies-Owen et al. (2024) reported that pleasant fragrances enhanced ratings of confidence, attractiveness, and femininity in faces, alongside corresponding changes in neural responses. One possible explanation is that facial expressions are perceived as social stimuli and engage more complex cognitive processes, possibly related to empathic states not captured in the present study (Rekow et al. 2021). Previous research using electroencephalography has found that the influence of odors on facial expressions occurs at late and ultra late latency periods of event-related potentials, reflecting higher levels of processing (Cook et al. 2015). Additionally, studies on how complex emotional stimuli, such as threats, were processed in the presence of faces indicated that at later stages of processing, faces can dominate over emotional content (Ferri et al. 2012).Together, this evidence might suggest that, during evaluation of faces with odors, cognitive or social aspects may contribute alongside individual emotional connections to the fragrance, although further research is needed to investigate this.

Comparison of emotional domains from LEOS in each of the odor conditions showed that lavender, rose, and vanilla are associated with enhanced happiness, nostalgia, relaxation, sensuality, and energy relative to clean air. This is consistent with Sayorwan et al. (2012), who investigated the mood and brain responses elicited by lavender using GEOS and electroencephalogram (EEG). They reported that lavender increased feelings of relaxation, happiness, energy, and freshness, which was also reflected in the corresponding EEG patterns (Sayorwan et al. 2012). On the other hand, lemon fragrance was only found to be associated with comparatively smaller effects in the domains of increased nostalgia, sensuality and energy compared to clear air. This difference may also explain the comparatively reduced impact of lemon fragrance on hedonic ratings of images relative to other fragrances. This finding is also in line with Hirama et al. (2025), who examined how different odors were perceived by Japanese people using an adapted version of the GEOS scale. They found that pleasant odors increased positive emotion ratings, whereas unpleasant odors were generally aligned to increased ratings of negative associations like disgust. Interestingly, disgust and thirstiness dimensions did not differ significantly across odors in the present study, despite disgust emerging as a significant covariate for hedonic evaluation of both landscapes and objects. This suggests that individual variability in disgust responses is important for evaluation of those images, despite not being a primary response for these generally pleasant fragrances.

The overall enhancement in ratings for objects by lavender, rose, and vanilla may be explained by the integration of the reward-processing regions in the brain linked to emotion and hedonic perception (Hummel et al. 1997; Low et al. 2021). Lemon was the only fragrance which did not show increased nostalgia LEOS ratings compared to clean air. Emotional states, such as nostalgia, are often linked to positive memories, triggering a “sense of reward” that may enhance the appreciation of a visual scene (Oba et al. 2016; Zhou and Kawabata 2025). The tight link between nostalgia, reward, and olfaction is well established, with physiological, psychological, and neurobiological evidence indicating that nostalgia is more frequently associated with positive than negative memories (Green et al. 2023). Moreover, this relationship may also involve other emotions such as happiness and sentimentality (Oba et al. 2016). Therefore, further studies are needed to better understand this emotion–odor–hedonism network and the role of subjective associations to nostalgia or odor memories.

### Limitations

The present research has several limitations. Firstly, the valence of the pictures was consistently high, which may indicate a ceiling effect, especially during the positive landscape pictures. When pleasantness ratings for pictures under the control (clean air) condition are already high, the opportunity for further odor-related enhancement is limited. This likely contributed to the lack of interaction between odors and image valence. As a result, this limitation may have constrained our ability to detect subtle fragrance-related modulations. To address this in future research, it will be important to consider visual stimuli with a broader range of pleasantness, alongside a wider variety of pleasant odors, to ensure sufficient variance for detecting cross-modal influences. A second limitation involves the inclusion of lemon fragrance. Despite the evidence drawn from the literature and the prescreening session to select the most suitable dilution, lemon was not consistently perceived as pleasant by the sample, and this might have influenced the effects of the other odors. This could be due to sample characteristics (e.g. the sample of our pilot might not have been representative of our study population) or the context-specific associations (e.g. lemon is often used in cleaning products) that might have affected its perceived pleasantness relative to other conditions. On the other hand, the relatively poorer performance of lemon for boosting of hedonic evaluations when compared (in a repeated-measures design) to other fragrances offers an interesting outcome which may be obscured by simple positive–negative or between-subjects designs typically employed in previous literature. Therefore, this outcome can also be seen as a strength of the current research design. More studies should consider individual differences in odor perception and the importance of contextual comparison between multiple fragrances included in study design in future.

Additionally, across both main analyses, odor effects were present but small in magnitude. Our sensitivity analysis showed that our study had 80% power to detect small-to-moderate effects (see Supplementary Appendix G). The null results in the present study might be consistent with very small effects rather than evidence of absence. However, several category-specific odor effects did emerge (within the interaction between odor and image type), suggesting that the study was sufficiently powered to detect nuanced odor effects. Future research using equivalence testing or Bayesian model comparison would help determine about whether these very small odor–valence effects are meaningfully absent or simply below the sensitivity of the current design.

### Conclusion

Our findings show that pleasant odors do exhibit the expected general effect of enhancing the evaluation of both positive and neutral pictures of faces and objects relative to clean air. However, differences across odor conditions indicate more nuanced patterns in hedonic evaluations of facial expressions and landscapes. We show, for the first time, that the impact of odors on hedonic judgments of images is associated with the emotional experience they elicit in individuals, and that this relationship varies across emotional dimensions, visual stimuli type, and the affective profile of the odor condition. This study has a wide range of applications, particularly for supporting future study design for investigations of odor effects on visual processing. Future research is needed to investigate the cortical networks underlying these cross-modal interactions, and how they are influenced by individual differences in emotional dimensions of odors.

## Supplementary Material

bjag027_Supplementary_Data

## Data Availability

Anonymized data along with analysis scripts can be found at the University of Liverpool data catalogue (DataCat). The data can be accessed using the following link: https://doi.org/10.17638/datacat.liverpool.ac.uk/3062.
