# Supplementary material for: Cross-modal impacts of olfactory perception and individual differences in emotional responses to odors modulate hedonic evaluations of visual scenes
Source: Chem Senses. 2026 Aug 25;51:bjag027. doi: 10.1093/chemse/bjag027 (PMC13547858; doi:10.1093/chemse/bjag027)
Supplement: Supplementary file 1 [file bjag027_supplementary_data.docx]

**Appendix A**

**Means rating scores of the Chicago Face Database (CFD)’s pictures (Ma et al., 2015)**

In table A1, for each face set, there is the list of the means for ratings of happiness, attractiveness, trustworthiness, and sadness extracted from the CFD’s manual (Ma et al., 2015). Moreover, in table A2, for each face image, there are the means for ratings of intensity and pleasantness from pre-experiment screening session we conducted to select the stimuli. We decided to choose as positive valence (happy expressions) the images showing open smiles (HO), to have the highest impact on valence and intensity.

**Table A1**

| *Means of happiness, attractiveness, trustworthiness, and sadness for the CFD’s images* | | | | | | |
| --- | --- | --- | --- | --- | --- | --- |
| Number | Sex | Ethnicity | Happy | Attractive | Trustworthy | Sad |
| BM-009 | Male | Black | 1.98 | 2.91 | 3.10 | 3.06 |
| WF-003 | Female | White | 3.33 | 4.89 | 4.05 | 2.36 |
| BF-044 | Female | Black | 2.16 | 2.10 | 3.26 | 3.04 |
| WF-030 | Female | White | 2.28 | 2.73 | 3.28 | 3.80 |
| WM-039 | Male | White | 2.45 | 3.36 | 3.67 | 2.83 |
| WF-013 | Female | White | 3.60 | 3.57 | 4.01 | 1.93 |
| WF-039 | Female | White | 2.45 | 3.36 | 3.70 | 2.83 |

*Note.* Number = The identification number in the dataset. Each face set is based on an actor or actress, and it has one image with neutral expression and one image with happy expression. The scores are based on a scale where 1 = Not at all, to 7 = Extremely (Ma et al., 2015).

**Table A2**

*Pilot means for intensity and pleasantness for the CFD’s images*

| CFD Number | Pilot ID | Sex | Ethnicity | Expression | Pleasantness (SD) | Intensity  (SD) |
| --- | --- | --- | --- | --- | --- | --- |
| BM-009-002-N | 1FN | Male | Black | Neutral | 2.91 (±2.55) | 3.23 (±2.47) |
| BM-009-003-HO | 1FH | Male | Black | Happy | 8.05 (±0.02) | 7.94 (±0.04) |
| WF-003-003-N | 2FN | Female | White | Neutral | 3.30 (±2.53) | 4.96 (±0.02) |
| WF-003-004-HO | 2FH | Female | White | Happy | 7.65 (±0.15) | 7.50 (±0.44) |
| BF-044-014-N | 4FN | Female | Black | Neutral | 4.07 (±1.27) | 4.65 (±0.47) |
| BF-044-009-HO | 4FH | Female | Black | Happy | 7.74 (±0.26) | 7.60 (±0.19) |
| WF-030-002-N | 5FN | Female | White | Neutral | 3.33 (±0.21) | 4.98 (±0.03) |
| WF-030-003-HO | 5FH | Female | White | Happy | 9.40 (±0.84) | 7.70 (±0.33) |
| WM-039-018-N | 6FN | Male | White | Neutral | 3.22 (±0.97) | 4.90 (±0.59) |
| WM-039-008-HO | 6FH | Male | White | Happy | 6.20 (±1.00) | 8.10 (±0.09) |
| WF-013-003-N | 7FN | Female | White | Neutral | 2.90 (±2.65) | 5.00 (±0.02) |
| WF-013-006-HO | 7FH | Female | White | Happy | 7.83 (±0.08) | 7.56 (±0.51) |
| WF-039-025-N | 8FN | Female | White | Neutral | 6.70 (±1.58) | 4.98 (±0.07) |
| WF-039-005-HO | 8FH | Female | White | Happy | 6.76 (±1.59) | 7.92 (±0.28) |

*Note.* CFD number = the identification number in the CFD dataset, Pilot ID = the number used in both pilot and study. The current scores were made on a 10-points scale, where 0 = Not at all to 10 = Very pleasant/intense.

**Appendix B**

**Means and standard deviations for valence and arousal of IAPS’s pictures (Lang et al., 2008)**

In table B1, for each picture of landscapes and objects, there is the list of means and standard deviations for valence and arousal extracted from the IAPS’s technical manual (Lang et al., 2008). Additionally, table B2 shows the means for the selected IAPS’s pictures based on intensity and pleasantness from the pre-experiment screening session.

**Table B1**

| *Means and standard deviations for valence and arousal of IAPS’s pictures* | | | | | | |
| --- | --- | --- | --- | --- | --- | --- |
| Item | Name | Number (IAPS) | Val mean | Val sd | Aro mean | Aro sd |
| Mug | 1ON | 7035 | 4.98 | 0.96 | 2.66 | 1.82 |
| Old Clock | 1OE | 7190 | 5.55 | 1.34 | 3.84 | 2.06 |
| Clock | 2ON | 7211 | 4.81 | 1.78 | 4.20 | 2.40 |
| Keyring | 3ON | 7059 | 4.93 | 0.81 | 2.73 | 1.88 |
| Ancient Vase | 2OE | 7192 | 5.77 | 1.33 | 3.58 | 2.01 |
| Puzzle | 3OE | 7061 | 5.40 | 1.40 | 3.66 | 1.92 |
| Clothespins | 5OE | 7052 | 5.33 | 1.32 | 3.01 | 2.02 |
| Stool | 5ON | 7025 | 4.63 | 1.17 | 2.71 | 2.20 |
| Cabinet | 6ON | 7705 | 4.77 | 1.02 | 2.65 | 1.88 |
| Book | 6OE | 7090 | 5.19 | 1.46 | 2.61 | 2.03 |
| Clothes Racket | 7ON | 7217 | 4.82 | 0.99 | 2.43 | 1.64 |
| Scarves | 7OE | 7205 | 5.56 | 1.39 | 2.93 | 2.16 |
| Basket | 8ON | 7010 | 4.94 | 1.07 | 1.76 | 1.48 |
| Suitcases | 8OE | 7081 | 5.36 | 1.30 | 3.96 | 2.24 |
| Bedroom | 2LN | 7710 | 5.42 | 1.58 | 3.44 | 2.21 |
| Desert Road | 3LN | 5900 | 5.93 | 1.64 | 4.38 | 2.10 |
| Station | 4LN | 7033 | 5.40 | 1.57 | 3.99 | 2.14 |
| Freeway | 5LN | 7560 | 4.47 | 1.65 | 5.24 | 2.03 |
| Rock Sea | 1LE | 5825 | 8.03 | 1.18 | 5.46 | 2.72 |
| Mountain | 2LE | 5600 | 7.57 | 1.48 | 5.19 | 2.70 |
| Skyline | 3LE | 7570 | 6.97 | 1.69 | 5.54 | 2.34 |
| Cruise | 5LE | 7492 | 7.41 | 1.68 | 4.91 | 2.46 |
| Traffic | 6LN | 7595 | 4.55 | 1.46 | 3.77 | 2.22 |
| Bridge | 7LN | 7547 | 5.21 | 0.96 | 3.18 | 2.01 |
| White House | 8LN | 7530 | 6.71 | 1.36 | 4.00 | 2.14 |
| Castle | 6LE | 7502 | 7.75 | 1.4 | 5.91 | 2.31 |
| Ferris Wheel | 7LE | 7508 | 7.02 | 1.46 | 5.09 | 2.11 |
| Seaside | 8LE | 5833 | 8.22 | 1.08 | 5.71 | 2.66 |

*Note.* Item= the item showed in the picture; Name= the name given in this study, with an identification number plus descriptive letters where O = object, L = landscape, N = neutral, E = emotional; Number (IAPS)= identification number of the picture in the IAPS system, Val mean= mean of the valence, Val sd= standard deviation of the valence, Aro mean= mean of the arousal, Aro sd= standard deviation of the arousal; ratings were made on a 9-points scale (Lang et al., 2008).

**Table B2**

*Means of IAPS’s pictures from pilot study*

| Item | Name | Number (IAPS) | Pleasantness (SD) | Intensity (SD) |
| --- | --- | --- | --- | --- |
| Mug | 1ON | 7035 | 4.99 (±0.03) | 4.98 (±0.04) |
| Old Clock | 1OE | 7190 | 6.04 (±0.76) | 6.20 (±0.38) |
| Clock | 2ON | 7211 | 6.22 (±1.43) | 5.05 (±0.02) |
| Keyring | 3ON | 7059 | 3.81 (±1.61) | 4.99 (±0.01) |
| Ancient Vase | 2OE | 7192 | 9.86 (±0.20) | 6.88 (±0.80) |
| Puzzle | 3OE | 7061 | 7.02 (±1.40) | 4.97 (±0.01) |
| Clothespins | 5OE | 7052 | 4.99 (±0.04) | 4.97 (±0.05) |
| Stool | 5ON | 7025 | 5.35 (±0.42) | 5.00 (±0.52) |
| Cabinet | 6ON | 7705 | 2.02 (±1.28) | 5.00 (± 0.01) |
| Book | 6OE | 7090 | 6.57 (±0.34) | 5.01 (±0.01) |
| Clothes Racket | 7ON | 7217 | 7.19 (±2.26) | 5.00 (±0.01) |
| Scarves | 7OE | 7205 | 6.21 (±0.14) | 5.11 (±0.17) |
| Basket | 8ON | 7010 | 4.98 (±0.06) | 1.23 (±0.62) |
| Suitcases | 8OE | 7081 | 6.15 (±1.46) | 1.41 (±0.08) |
| Bedroom | 2LN | 7710 | 8.75 (±0.94) | 3.84 (±1.63) |
| Desert Road | 3LN | 5900 | 5.43 (±2.49) | 3.29 (±0.70) |
| Station | 4LN | 7033 | 4.46 (±2.15) | 4.82 (±0.16) |
| Freeway | 5LN | 7560 | 8.04 (±1.86) | 3.75 (±0.10) |
| Rock Sea | 1LE | 5825 | 8.42 (±0.54) | 8.29 (±0.58) |
| Mountain | 2LE | 5600 | 8.53 (±1.04) | 8.08 (±0.68) |
| Skyline | 3LE | 7570 | 6.97 (±2.59) | 7.93 (±0.14) |
| Cruise | 5LE | 7492 | 9.45 (±0.06) | 8.06 (±0.29) |
| Traffic | 6LN | 7595 | 5.65 (±2.72) | 4.89 (±0.20) |
| Bridge | 7LN | 7547 | 7.22 (±1.28) | 4.97 (±0.01) |
| White House | 8LN | 7530 | 5.43 (±0.03) | 1.73 (±0.26) |
| Castle | 6LE | 7502 | 7.21 (±1.79) | 8.05 (±0.18) |
| Ferris Wheel | 7LE | 7508 | 6.52 (±0.83) | 8.15 (±0.03) |
| Seaside  *Note.* Item = the item showed in the picture; Name= the name given in this study, with an identification number plus descriptive letters where O = object, L = landscape, N = neutral, E = emotional; Number (IAPS)= identification number of the picture in the IAPS system, SD = standard deviation. | 8LE | 5833 | 7.97 (±2.20) | 0.77 (±0.12) |

**Appendix C**

**Extracted examples from literature of the use of the chosen odours**

In table C1 there are some extracted example from the literature showing that the odours chosen for this study are the most common.

N.B. the current literature review was focused only on neuroimaging studies involving olfaction and emotions

**Table C1**

*Extracted example from literature review of the current odours used*

| Title | Year | Authors | Summary aims | Odour used |
| --- | --- | --- | --- | --- |
| Psychophysiological Responses of Cut Flower Fragrances as an Olfactory Stimulation by Measurement of Electroencephalogram in Adults. | 2022 | Wu, Yu-Tong and Lee, A-Young and Choi, Na-Yoon and Park, Sin-Ae | The objective of this study was set to investigate the psychophysiological responses of individuals participating in FA using different flowers. | Flower scents like **rose** and lily |
| Pleasantness Recognition Induced by Different Odor Concentrations Using Olfactory Electroencephalogram Signals. | 2022 | Hou, Hui-Rang and Han, Rui-Xue and Zhang, Xiao-Nei and Meng, Qing-Hao | To explores pleasantness recognition induced by different odor concentrations for the first time. | **Rose** was used as pleasant condition |
| OVPD: Odor-Video Elicited Physiological Signal Database for Emotion Recognition | 2022 | Xue, JY and Wang, JQ and Hu, S and Bi, N and Lv, Z | The motivation for their research was to determine a subject’s  changes in emotional perception of a video modulated by odor  using the OVPD. The major contribution of this article can  be summarized | **Rose** was one of the odour conditions |
| Neural Processing of Odors with Different Well-Being Associations-Findings from Two Consecutive Neuroimaging Studies. | 2023 | Joshi A and Hornstein H and Thaploo D and Faria V and Warr J and Hummel T | They performed two consecutive studies in two distinct populations to understand how humans process odors with different WB valence | **Rose, lavender,** and **vanilla** were some of the odours used |
| EEG asymmetry responses to lavender and rosemary aromas in adults and infants. | 2022 | Sanders, Chris and Diego, Miguel and Fernandez, Mercedes and Field, Tiffany and Hernandez-Reif, Maria and Roca, Amparo | This study examined frontal asymmetry in adults during lavender or rosemary exposure. Rosemary was used as a control group stimulus because no empirically based mood effects have been reported for rosemary. | **Lavender** vs rosemary |
| Sustained effects of pleasant and unpleasant smells on resting state brain activity | 2020 | Carlson, H and Leitao, J and Delplanque, S and Cayeux, I and Sander, D and Vuilleumier, P | They tested whether smells could also evoke powerful lingering effects on brain activity subsequent to odorant exposure, persisting even during resting state when participants have no task to perform | **Lavender** was one of the pleasant odours |
| Evaluation of human electroencephalogram change for sensory effects of fragrance | 2019 | Park, K.H. and Kim, H.J. and Oh, B. and Seo, M. and Lee, E. and Ha, J. | This study aims to create a new emotional cosmetic evaluation paradigm using EEG values | **Lavender** was one of the odour conditions |
| Modulation of cerebral haemodynamic response to olfactory stimuli by emotional valence detected by functional magnetic resonance imaging | 2015 | Caous, C.A. and Tobo, P.R. and Talarico, V.H. and GonÃ§ales, L.R.L. and Yoshimine, E. and Da Cruz, A.C., Jr. and Albuquerque, C. and Amaro, E., Jr. | The olfactory paradigm was designed to analyze brain response to single odorants with clear emotional components, allowing the relationship between emotional valence and hemodynamic response of the brain to be studied | **Lavender** was the pleasant condition |
| Can ambient odors influence the recognition of emotional words? A behavioral and event-related potentials study | 2022 | Li, Danyang and Wang, Xiaochun | To explore whether different odor contexts can help people identify emotions, thereby demonstrating whether odor environments can facilitate social interactions. Identify behavioural and neural mechanisms of the effect of smell on visual processing of emotional words. | **Vanilla** and **lemon** were two of the odours used |
| Odor-induced emotion recognition based on average frequency band division of EEG signalDifferent Brain Activation in Response to Repeated Odors of Pleasantness and Unpleasantnesss | 2020 | Hou, HR and Zhang, XN and Meng, QH | They focused on classifying emotions during the perception of thirteen odors. To do so, power-spectral-density (PSD) features were extracted based on equal frequency bandwidths, rather than the traditional EEG rhythm-based bandwidth | **Vanilla, lemon, lavender,** and **rose** were involved |
| Impact of valence and age on olfactory induced brain activation in healthy women | 2010 | Reske, Martina and Kellermann, Thilo and Shah, N. Jon and Schneider, Frank and Habel, Ute | They aimed to determine the influence of age on cerebral odor-induced activations assuming that fMRI is sensitive enough to identify early age-related changes in olfactory processing not already detectable in behavioral studies | **Vanilla** was used as control |
| Anterior electroencephalographic asymmetry changes in elderly women in response to a pleasant and an unpleasant odor | 2000 | Kline, John P. and Blackhart, Ginette C. and Woodward, Kathrane M. and Williams, Sherry R. and Schwartz, Gary E. R. | The present study examined changes in anterior asymmetry with a pleasant, unpleasant, and a neutral odor. The authors were specifically interested in: (1) whether a pleasant odor (vanilla) would result in left frontal activation; and (2) whether an unpleasant odor (valerian) would result in right frontal activation. | **Vanilla** as pleasant odour |
| Forefront users experience evaluation by employing together virtual reality and electroencephalography: A case study on cognitive effects of scents | 2021 | Mancini, M. and Cherubino, P. and Cartocci, G. and Martinez, A. and Borghini, G. and Guastamacchia, E. and Flumeri, G.D. and Rossi, D. and Modica, E. and Menicocci, S. and Lupo, V. and Trettel, A. and Babiloni, F. | The present work aimed at investigating the feasibility and capability in using VR and EEG to investigate users’ experiences with respect to the particular scents vaporized. | **Lemon** and **lavender** were the only two odours. |
| Unpleasant Food Odors Modulate the Processing of Facial Expressions: An Event-Related Potential Study | 2020 | Li, D. and Jia, J. and Wang, X. | The current research on the integration of facial expressions and olfactory emotional information reveals two phenomena. One is that olfactory emotional information will affect the evaluation and judgment of neutral or ambiguous facial expressions. Another is that odor will modulate the processing of facial expressions, but it is unclear whether odor-based modulations occur only for emotionally congruent olfactory-visual stimuli or whether odor has an overall effect on the processing of facial expression. | **Lemon** was used as pleasant food odour |
| Integrated neural representations of odor intensity and affective valence in human amygdala. | 2005 | Winston, Joel S and Gottfried, Jay A and Kilner, James M and Dolan, Raymond J | They reasoned that intensity-evoked responses within the amygdala might depend on the level of valence. On theoretical grounds, they also predicted that amygdala activation might show a reliance on valence extremes, given that chemosensory strength or intensity conceivably takes on greater importance when the stimulus is pleasant or unpleasant. | **Lemon** was one of the pleasant conditions |

*Note.* This is only a small list of examples to prove how the odours used in the current study are common in the olfactory literature.

**Appendix D**

**Odours’ dilutions selection**

In table D1 there is the mean ratings for pleasantness and intensity of each odour’s dilution based on our pilot study, conducted to select each stimulus.

**Table D1**

*Pilot means of pleasantness and intensity of each odour’s dilution*

| Odour | Dilution | Pleasantness (SD) | Intensity (SD) | Selected? |
| --- | --- | --- | --- | --- |
| Lemon | 0.50% | 6.43 (±0.98) | 6.28 (±1.38) | X |
| Lemon | 1.00% | 5.71 (±1.70) | 7.00 (±1.53) | X |
| Lemon | 0.20% | 6.00 (±1.15) | 4.57 (±2.51) | X |
| Lemon | 0.10% | 5.71 (±1.25) | 5.29 (±2.50) | X |
| Lemon | 0.05% | 6.00 (±2.31) | 4.29 (±2.14) | Chosen |
| Vanilla | 2.00% | 7.86 (±1.07) | 6.00 (±1.53) | Chosen |
| Vanilla | 1.00% | 7.00 (±1.29) | 5.29 (±2.14) | X |
| Vanilla | 0.50% | 7.57 (±1.72) | 6.57 (±1.40) | X |
| Rose | 0.20% | 5.74 (±1.38) | 5.43 (±2.25) | X |
| Rose | 2.00% | 5.71 (±1.25) | 7.14 (±2.27) | X |
| Rose | 0.50% | 6.00 (±1.15) | 4.29 (±1.80) | Chosen |
| Lavender | 0.50% | 6.29 (±1.80) | 5.86 (±2.04) | X |
| Lavender | 0.20% | 6.57 (±0.79) | 4.71 (±1.98) | Chosen |
| Lavender | 0.10% | 6.14 (±2.12) | 6.43 (±2.30) | X |
| Lavender | 0.05% | 5.50 (±2.14) | 3.71 (±2.56) | X |

*Note.* SD = standard deviation. The current table shows the mean ratings for intensity and pleasantness, with 0 = Not at all, and 10 = very pleasant/intense. We selected one dilution for each odour based on the best pleasantness:intensity ratio.

**Appendix E**

**Descriptive statistics for image ratings**

In table E1 there is the descriptive statistics for subjective ratings across all the types of images based on odours and valence.

**Table E1**

| *Descriptive statistics for image’s ratings based on type, odour, and valence* | | | | | | |
| --- | --- | --- | --- | --- | --- | --- |
| Type | Odour | Valence | Mean | SD | Min | Max |
| Faces | Air | Neutral | 2.32 | 1.87 | 0.00 | 6.38 |
|  |  | Positive | 4.52 | 2.52 | 0.00 | 9.95 |
|  | Lavender | Neutral | 2.55 | 1.80 | 0.00 | 6.53 |
|  |  | Positive | 4.70 | 2.57 | 0.00 | 9.84 |
|  | Lemon | Neutral | 2.31 | 1.89 | 0.00 | 6.74 |
|  |  | Positive | 4.15 | 2.48 | 0.00 | 10.00 |
|  | Rose | Neutral | 2.47 | 1.88 | 0.00 | 6.93 |
|  |  | Positive | 4.47 | 2.53 | 0.00 | 9.97 |
|  | Vanilla | Neutral | 2.49 | 1.80 | 0.00 | 6.79 |
|  |  | Positive | 4.54 | 2.71 | 0.00 | 10.00 |
| Landscapes | Air | Neutral | 3.80 | 2.88 | 0.00 | 10.00 |
|  |  | Positive | 7.69 | 1.72 | 3.40 | 10.00 |
|  | Lavender | Neutral | 3.94 | 2.77 | 0.00 | 10.00 |
|  |  | Positive | 7.70 | 1.78 | 3.33 | 10.00 |
|  | Lemon | Neutral | 3.58 | 2.82 | 0.00 | 10.00 |
|  |  | Positive | 7.15 | 1.91 | 3.17 | 10.00 |
|  | Rose | Neutral | 4.03 | 2.82 | 0.00 | 10.00 |
|  |  | Positive | 7.69 | 1.66 | 3.56 | 10.00 |
|  | Vanilla | Neutral | 4.04 | 2.90 | 0.00 | 10.00 |
|  |  | Positive | 7.80 | 1.77 | 3.40 | 10.00 |
|  | Air | Neutral | 1.54 | 1.43 | 0.00 | 5.04 |
|  |  | Positive | 3.03 | 2.26 | 0.00 | 8.52 |
|  | Lavender | Neutral | 1.89 | 1.57 | 0.00 | 5.49 |
| Objects |  | Positive | 3.44 | 2.37 | 0.00 | 8.95 |
|  | Lemon | Neutral | 1.44 | 1.26 | 0.00 | 4.56 |
|  |  | Positive | 3.01 | 2.13 | 0.00 | 8.23 |
|  | Rose | Neutral | 1.85 | 1.56 | 0.00 | 5.44 |
|  |  | Positive | 3.36 | 2.37 | 0.00 | 8.96 |
|  | Vanilla | Neutral | 1.98 | 1.66 | 0.00 | 5.38 |
|  |  | Positive | 3.35 | 2.36 | 0.00 | 8.83 |

**Appendix F**

**Descriptive statistics for LEOS ratings**

Table F1 shows the descriptive statistics for each emotional domain rating from LEOS based on each odour

**Table F1**

|  | *Descriptive statistics for the LEOS ratings based on each odour* | | | | | | |
| --- | --- | --- | --- | --- | --- | --- | --- |
| Dimension | | Odour | | Mean | SD | Min | Max |
| Happiness | | Air | | 3.25 | 2.54 | 0.00 | 10.00 |
|  |  | Lavender | | 5.37 | 2.49 | 0.00 | 9.99 |
|  |  | Lemon | | 4.36 | 2.55 | 0.00 | 9.87 |
|  |  | Rose | | 4.97 | 2.61 | 0.00 | 10.00 |
|  |  | Vanilla | | 5.44 | 2.82 | 0.00 | 10.00 |
|  | Nostalgia | | Air | 2.03 | 2.51 | 0.00 | 9.98 |
|  |  |  | Lavender | 4.61 | 2.94 | 0.00 | 9.99 |
|  |  |  | Lemon | 3.55 | 2.86 | 0.00 | 10.00 |
|  |  |  | Rose | 4.98 | 2.77 | 0.03 | 9.99 |
|  |  |  | Vanilla | 4.33 | 3.05 | 0.00 | 10.00 |
|  | Disgust | | Air | 0.99 | 2.05 | 0.00 | 9.93 |
|  |  |  | Lavender | 1.54 | 2.69 | 0.00 | 10.00 |
|  |  |  | Lemon | 2.10 | 2.82 | 0.00 | 9.95 |
|  |  |  | Rose | 1.47 | 2.22 | 0.00 | 9.89 |
|  |  |  | Vanilla | 1.41 | 2.55 | 0.00 | 9.92 |
|  | Relaxation | | Air | 4.60 | 2.78 | 0.00 | 10.00 |
|  |  |  | Lavender | 6.58 | 2.59 | 0.00 | 9.99 |
|  |  |  | Lemon | 4.47 | 2.75 | 0.00 | 10.00 |
|  |  |  | Rose | 5.80 | 2.41 | 0.97 | 10.00 |
|  |  |  | Vanilla | 5.85 | 2.74 | 1.01 | 9.97 |
|  | Sensuality | | Air | 1.18 | 2.14 | 0.00 | 9.97 |
|  |  |  | Lavender | 2.87 | 2.63 | 0.00 | 10.00 |
|  |  |  | Lemon | 2.05 | 2.49 | 0.00 | 10.00 |
|  |  |  | Rose | 2.64 | 2.66 | 0.00 | 9.99 |
|  |  |  | Vanilla | 3.91 | 3.35 | 0.00 | 9.96 |
|  | Thirstiness | | Air | 3.23 | 3.44 | 0.00 | 10.00 |
|  |  |  | Lavender | 3.04 | 2.78 | 0.00 | 9.01 |
|  | Thirstiness | | Lemon | 3.29 | 2.82 | 0.00 | 9.96 |
|  |  |  | Rose | 2.50 | 2.12 | 0.00 | 7.66 |
|  |  |  | Vanilla | 3.02 | 2.92 | 0.00 | 10.00 |
|  | Energy | | Air | 1.30 | 1.85 | 0.00 | 6.79 |
|  |  |  | Lavender | 3.44 | 2.33 | 0.00 | 8.57 |
|  |  |  | Lemon | 4.15 | 3.00 | 0.00 | 9.92 |
|  |  |  | Rose | 2.79 | 2.21 | 0.00 | 7.80 |
|  |  |  | Vanilla | 2.91 | 2.72 | 0.00 | 9.22 |

*Note*. SD = Standard deviation, Min = minimum, Max = maximum

**Appendix G**

**Extra analyses: correlations and power analysis**

***Correlations among LEOS covariates***

Table G1 presents the Pearson correlation matrix for the LEOS emotional dimensions. This analysis was conducted to assess the degree of shared variance among covariates and to check for potential multicollinearity prior to inclusion in the mixed-effect ANCOVA model.

**Table G1**

| *Correlation matrix for LEOS emotional dimensions* | | | | | | | |
| --- | --- | --- | --- | --- | --- | --- | --- |
|  | Happiness | Nostalgia | Sensuality | Disgust | Relaxation | Thirstiness | Energy |
| Happiness | 1.00 | .48 | .47 | -.10 | .53 | .06 | .53 |
| Nostalgia | - | 1.00 | .49 | .10 | .35 | .07 | .45 |
| Sensuality | - | - | 1.00 | .22 | .34 | .07 | .37 |
| Disgust | - | - | - | 1.00 | .19 | .13 | .10 |
| Relaxation | - | - | - | - | 1.00 | .09 | .18 |
| Thirstiness | - | - | - | - | - | 1.00 | .07 |

*Note.* All correlations are Pearson’s r. Values below the diagonal are omitted for clarity.

***Power sensitivity analysis***

A sensitivity analysis was conducted using R studio 2023.12.1 (Rstudio Team, USA) with the pwr package (Champely, 2020). With 40 participants, α =.05, and five within-subject odour conditions, the design had 80% power to detect effects of at least *f* = 0.25, corresponding to approximately $\eta_{p}^{2}$ = .06.

**Appendix H**

**Model estimates for model of image pleasantness rating and for the LEOS model**

Table H1 presents the estimates of fixed and random effects from the mixed-effects model of image pleasantness ratings. Table H2 presents the fixed and random effects estimates from the mixed-effects model with LEOS covariates for image pleasantness ratings.

**Table H1**

| *Full mixed-effects model estimates for image pleasantness* | | | | | |
| --- | --- | --- | --- | --- | --- |
| Fixed Effects | | | | | |
|  | Est/Beta | SE | 95% CI | *t* | p |
| Intercept | 1.98 | 0.35 | 1.31 – 2.67 | 5.65 | 0.0000001 |
| Odour  Lavender | 0.32 | 0.18 | -0.03 – 0.66 | 1.79 | 0.0734 |
| Odour  Lemon | -0.01 | 0.18 | -0.35 – 0.34 | -0.034 | 0.9739 |
| Odour  Rose | 0.12 | 0.18 | -0.22 – 0.47 | 0.690 | 0.4901 |
| Odour  Vanilla | 0.33 | 0.18 | -0.02 – 0.67 | 1.850 | 0.0644 |
| Valence  Positive | 1.60 | 0.40 | 0.84 – 2.39 | 4.048 | 0.0000853 |
| Type  Landscape | 2.26 | 0.40 | 1.46 – 3.02 | 5.728 | 0.0000000583 |
| Type  Object | -0.15 | 0.40 | -0.92 – 0.61 | -0.375 | 0.7085 |
|  | | | | | |
| Lavender x Positive | -0.12 | 0.25 | -0.61 – 0.37 | -0.482 | 0.6295 |
| Lemon x Positive | -0.25 | 0.25 | -0.74 – 0.24 | -0.990 | 0.3222 |
| Rose x Positive | -0.20 | 0.25 | -0.69 – 0.29 | -0.813 | 0.4164 |
| Vanilla x Positive | -0.25 | 0.25 | -0.74 – 0.24 | -0.987 | 0.3239 |
|  | | | | | |
| Lavender x Landscape | -0.42 | 0.25 | -0.91 – 0.07 | -1.683 | 0.0924 |
| Lemon x Landscape | -0.28 | 0.25 | -0.77 – 0.21 | -1.112 | 0.2662 |
| Rose x Landscape | 0.06 | 0.25 | -0.43 – 0.55 | 0.235 | 0.8144 |
| Vanilla x Landscape | -0.12 | 0.25 | -0.61 – 0.37 | 0.470 | 0.6387 |
| Lavender x Object | 0.07 | 0.25 | -0.42- 0.55 | 0.289 | 0.7726 |
| Lemon x Object | 0.18 | 0.25 | -0.31 – 0.66 | 0.708 | 0.4787 |
| Rose x Object | 0.30 | 0.25 | -0.19 – 0.79 | 1.191 | 0.2336 |
| Vanilla x Object | 0.23 | 0.25 | -0.26 – 0.72 | 0.920 | 0.3578 |
|  | | | | | |
| Positive x Landscape | 3.07 | 0.56 | 1.83 – 4.19 | 5.507 | 0.000000165 |
| Positive x Object | -0.50 | 0.56 | -1.59 – 0.57 | -0.889 | 0.3753 |
|  | | | | | |
| Lavender x Positive x Landscape | 0.01 | 0.35 | -0.68 – 0.70 | 0.033 | 0.9736 |
| Lemon x Positive x Landscape | -0.31 | 0.35 | -1.00 – 0.38 | -0.883 | 0.3774 |
| Rose x Positive x Landscape | -0.07 | 0.35 | -0.76 – 0.62 | -0.204 | 0.8381 |
| Vanilla x Positive x Landscape | 0.05 | 0.35 | -0.65 – 0.62 | 0.131 | 0.8960 |
| Lavender x Positive x Object | -0.06 | 0.35 | -0.76 – 0.63 | -0.180 | 0.8574 |
| Lemon x Positive x Object | 0.04 | 0.35 | -0.66 – 0.73 | 0.101 | 0.9199 |
| Rose x Positive x Object | 0.04 | 0.35 | -0.65 – 0.73 | 0.104 | 0.9170 |
| Vanilla x Positive x Object | -0.01 | 0.35 | -0.70 – 0.68 | -0.024 | 0.9808 |
|  | | | | | |
| Random Effects | | | | | |
|  | | | Variance | S.D. | |
| Image (Intercept) | | | 1.08 | 1.01 | |
| Participant (Intercept) | | | 1.22 | 1.11 | |
| Residuals | | | 4.37 | 2.09 | |
|  | | | | | |
| Model fit | | | | | |
| R^2^ | | | Marginal | Conditional | |
|  | | | 0.31 | 0.72 | |

*Note.* Model: Ratings ~ Odour * Valence * Type + (1|Participant) + (1|Image). Clean air, neutral and face were considered as baseline categories.

**Table H2**

| *Full mixed-effects model estimates for the LEOS model* | | | | | |
| --- | --- | --- | --- | --- | --- |
| Fixed Effects | | | | | |
|  | Est/Beta | SE | 95% CI | *t* | p |
| Intercept | 1.82 | 0.35 | 1.11 – 2.51 | 5.21 | 0.000000914 |
| Odour  Lavender | 0.17 | 0.18 | -0.18 – 0.52 | 0.96 | 0.338554 |
| Odour  Lemon | 0.01 | 0.18 | -0.35 – 0.36 | 0.05 | 0.960163 |
| Odour  Rose | -0.03 | 0.18 | -0.39 – 0.31 | -0.21 | 0.831164 |
| Odour  Vanilla | 0.13 | 0.18 | -0.22 – 0.48 | 0.72 | 0.473533 |
| Valence  Positive | 1.62 | 0.39 | 0.83 – 2.38 | 4.22 | 0.0000461 |
| Type  Landscape | 2.25 | 0.38 | 1.50 – 3.05 | 5.89 | 0.0000000302 |
| Type  Object | -0.16 | 0.38 | -0.91 – 0.62 | -0.40 | 0.689805 |
| Happiness | 0.05 | 0.02 | 0.02 – 0.08 | 3.54 | 0.000405 |
| Nostalgia | 0.06 | 0.01 | 0.04 – 0.08 | 5.30 | 0.000000117 |
| Disgust | -0.14 | 0.02 | -0.17 – -0.10 | -8.58 | < 2e-16 |
| Relaxation | -0.03 | 0.01 | -0.05 – 0.03 | -1.94 | 0.052379 |
| Sensuality | 0.02 | 0.02 | 0.01 – 0.05 | 1.47 | 0.142890 |
| Thirstiness | 0.04 | 0.01 | 0.01 – 0.06 | 2.74 | 0.006203 |
| Energy | -0.02 | 0.02 | -0.05 – 0.02 | -0.99 | 0.322202 |
|  | | | | | |
| Lavender x Positive | -0.12 | 0.25 | -0.60 – 0.36 | -0.49 | 0.624150 |
| Lemon x Positive | -0.25 | 0.25 | -0.73 – 0.23 | -1.01 | 0.314613 |
| Rose x Positive | -0.20 | 0.25 | -0.69 – 0.28 | -0.82 | 0.409946 |
| Vanilla x Positive | -0.25 | 0.25 | -0.73 – 0.24 | -1.00 | 0.316322 |
|  | | | | | |
| Lavender x Landscape | -0.42 | 0.25 | -0.90 – 0.06 | -1.71 | 0.087092 |
| Lemon x Landscape | -0.28 | 0.25 | -0.76– 0.20 | -1.13 | 0.257997 |
| Rose x Landscape | 0.06 | 0.25 | -0.42 – 0.54 | 0.24 | 0.812442 |
| Vanilla x Landscape | -0.12 | 0.25 | -0.60 – 0.36 | -0.48 | 0.632180 |
| Lavender x Object | 0.07 | 0.25 | -0.41 – 0.55 | -0.63 | 0.769092 |
| Lemon x Object | 0.18 | 0.25 | -0.31 – 0.66 | 0.72 | 0.470982 |
| Rose x Object | 0.30 | 0.25 | -0.18 – 0.78 | 1.21 | 0.226099 |
| Vanilla x Object | 0.23 | 0.25 | -0.25 – 0.71 | 0.93 | 0.350274 |
|  | | | | | |
| Positive x Landscape | 2.98 | 0.54 | 1.86 – 4.20 | 5.52 | 0.000000173 |
| Positive x Object | -0.51 | 0.54 | -1.58 – 0.57 | -0.95 | 0.346177 |
|  | | | | | |
| Lavender x Positive x Landscape | 0.01 | 0.35 | -0.67 – 0.69 | 0.04 | 0.970691 |
| Lemon x Positive x Landscape | -0.31 | 0.35 | -0.99 – 0.37 | -0.89 | 0.371655 |
| Rose x Positive x Landscape | -0.07 | 0.35 | -0.75 – 0.61 | -0.21 | 0.837142 |
| Vanilla x Positive x Landscape | 0.05 | 0.35 | -0.63 – 0.73 | 0.14 | 0.891872 |
| Lavender x Positive x Object | -0.06 | 0.35 | -0.75 – 0.62 | -0.18 | 0.855225 |
| Lemon x Positive x Object | 0.04 | 0.35 | -0.65 – 0.72 | 0.10 | 0.919872 |
| Rose x Positive x Object | 0.04 | 0.35 | -0.65 – 0.72 | 0.11 | 0.916611 |
| Vanilla x Positive x Object | -0.01 | 0.35 | -0.69 – 0.67 | 0.35 | 0.980542 |
|  | | | | | |
| Random Effects | | | | | |
|  | | | Variance | S.D. | |
| Image (Intercept) | | | 0.91 | 0.96 | |
| Participant (Intercept) | | | 1.24 | 1.12 | |
| Residuals | | | 4.24 | 2.06 | |
|  | | | | | |
| Model fit | | | | | |
| R^2^ | | | Marginal | Conditional | |
|  | | | 0.32 | 0.73 | |

*Note.* Model: Ratings ~ Odour * Valence * Type + Happiness + Nostalgia + Disgust + Relaxation + Sensuality + Thirstiness + Energy+ (1|Participant) + (1|Image). Clean air, neutral and face were considered as baseline categories.

**Appendix I**

**Post-hoc pairwise comparisons for LEOS ratings**

Post-hoc pairwise exploratory comparisons revealed that happiness ratings in presence of lavender (*t*(38) = 4.20, *p* < .001, Cohen’s *d* = 0.89, 95% CI [1.12, 3.22]), rose (*t*(38) = 2.87, *p* = .006, Cohen’s *d* = 0.61, 95% CI [0.46, 2.66]), and vanilla (*t*(38) = 3.28, *p* = .002, Cohen’s *d* = 0.86, 95% CI [0.86, 3.62]) were significantly higher compared to clean air. Moreover, lavender (*t*(38) = 3.32, *p* = .002, Cohen’s *d* = 0.50, 95% CI [0.48, 1.98]) and vanilla (*t*(38) = 2.89, *p* = .006, Cohen’s *d* = 0.50, 95% CI [0.39, 2.21]) further exceeded lemon. Nostalgia ratings were significantly higher during lavender (*t*(38) = 4.61, *p* < .001, Cohen’s *d* = 0.98, 95% CI [1.48, 3.81]), lemon (*t*(38) = 3.00, *p* = .005, Cohen’s *d* = 0.50, 95% CI [0.43, 2.19]), rose (*t*(38) = 5.50, *p* < .001, Cohen’s *d* = 1.07, 95% CI [1.78, 3.85]) , vanilla (*t*(38) = 3.57, *p* < .001, Cohen’s *d* = 0.77, 95% CI [0.91, 3.31]) compared to clean air. Moreover, lavender (*t*(38) = 2.48, *p* = .018, Cohen’s *d* = 0.48, 95% CI [0.25, 2.42]) and rose (*t*(38) = 2.79, *p* = .008, Cohen’s *d* = 0.55, 95% CI [0.41, 2.59]) further exceeded lemon.

For relaxation, lavender (*t*(38) = 3.88, *p* < .001, Cohen’s *d* = 0.80, 95% CI [0.98, 3.11]) and vanilla (*t*(38) = 2.11, *p* = .042, Cohen’s *d* = 0.46, 95% CI [0.05, 2.45]) enhanced ratings compared to clean air. Moreover, lavender (*t*(38) = 4.96, *p* < .001, Cohen’s *d* = 0.85, 95% CI [1.28, 3.05]), rose (*t*(38) = 2.35, *p* = .024, Cohen’s *d* = 0.46, 95% CI [0.16, 2.18]), and vanilla (*t*(38) = 2.90, *p* = .006, Cohen’s *d* = 0.51, 95% CI [0.42, 2.33]) further exceeded lemon. For sensuality, ratings were higher in presence of lavender (*t*(38) = 4.90, *p* < .001, Cohen’s *d* = 0.71, 95% CI [1.02, 2.46]), lemon (*t*(38) = 2.40, *p* = .021, Cohen’s *d* = 0.38, 95%CI [0.14, 1.65]), rose (*t*(38)= 4.55, *p* < .001, Cohen’s *d* = 0.61, 95% CI [0.83, 2.17]), and vanilla (*t*(38) = 5.34, *p* < .001, Cohen’s *d* = 0.97, 95% CI [1.74, 3.86]) compared to clean air. Furthermore, lavender (*t*(38) = 2.33, *p* = .025, Cohen’s *d* = 0.33, 95%CI [0.11, 1.58]) vanilla (*t*(38) = 3.97, *p* < .001, Cohen’s *d* = 0.63, 95% CI [0.93, 2.88]) further exceeded lemon. Interestingly, vanilla also exceeded lavender (*t*(38) = 2.11, *p* = .042, Cohen’s *d* = 0.35, 95% CI [0.04, 2.08]) and rose (*t*(38) = 2.63, *p* = .012, Cohen’s *d* = 0.43, 95% CI [0.30, 2.30]). Finally, for energy, lavender (*t*(38) = 4.86, *p* < .001, Cohen’s *d* = 1.05, 95% CI [1.28, 3.11]), lemon (*t*(38) = 5.34, *p* < .001, Cohen’s *d* = 1.08, 95% CI [1.70, 3.77]), rose (*t*(38) = 3.38, *p* = .002, Cohen’s *d* = 0.67, 95% CI [0.54, 2,14]), and vanilla *t*(38) = 3.04, *p* = .004, Cohen’s *d* = 0.63, 95% CI [0.48, 2.39]) elicited higher ratings compared to clean air. Moreover, lemon exceeded rose (*t*(38) = 3.39, *p* = .002, Cohen’s *d* = 0.52, 95% CI [0.56, 2.22]) and vanilla (*t*(38) = 2.79, *p* = .008, Cohen’s *d* = 0.46, 95% CI [0.35, 2.24]) Interestingly, lavender also exceeded rose (*t*(38) = 2.22, *p* = .033, Cohen’s *d* = 0.39, 95% CI [0.07, 1.64]). All other pairwise comparisons were non-significant (*p* > .05)

**Appendix J**

**Complementary analyses without the lemon odour condition**

***Mixed-effects model for image pleasantness ratings***

Without the lemon odour condition, the linear mixed-effect model revealed a main effect of image valence (*F*(1, 60.9) = 121.33, *p* < .001, $\eta_{p}^{2}$ = 0.67), with positive images rated as more pleasant than neutral images. Moreover, there were a significant main effect of odour (*F*(3, 6586.5) = 3.90, *p* =.009, $\eta_{p}^{2}$< 0.01), and of image type (*F*(2, 58.7) = 126.16, *p* < .001, $\eta_{p}^{2}$ = 0.81). The interaction between odour and image type was also significant (*F*(6, 6586.5) =2.11, *p* =.049, $\eta_{p}^{2}$ < 0.01), suggesting that odour effects varied across faces, landscapes, and objects. Exploratory follow-up pairwise comparisons showed that faces were rated significantly more pleasant in the presence of lavender compared to the clean air condition (*t*(6586) = 2.04, *p* = .041, Cohen’s *d* = 0.12, 95% CI [0.01, 0.50]). No other odour was significantly increasing ratings for faces compared to clean air (*p*> .05). Furthermore, the results showed that landscapes were rated significantly more pleasant during vanilla (*t*(6586) = 2.13, *p* = .033, Cohen’s *d* = 0.13, 95% CI [0.02, 0.51]) compared to lavender. The pairwise comparisons also showed that objects were rated significantly more pleasant during lavender *t*(6586) = 2.36, *p* = .018, Cohen’s *d* = 0.14, 95% CI [0.05, 0.54]), rose (*t*(6586) = 2.68, *p* = .007, Cohen’s *d* = 0.16, 95% CI [0.09, 0.58]), and vanilla (*t*(6586) = 3.42, *p* < .001, Cohen’s *d* = 0.20, 95% CI [0.18, 0.67]) compared to clean air.

Additionally, the interaction between image valence and image type was significant (*F*(2,61) = 21.47, *p* < .001, $\eta_{p}^{2}$ = 0.41), indicating that the effect of valence differed across image categories. Exploratory follow-up pairwise comparisons showed that positive pictures were rated significantly higher than neutral in landscapes (*t*(64.2) = 11.77, *p* < .001, Cohen’s *d* = 1.96, 95% CI [3.41, 4.81]), in faces (*t*(61.2) = 4.41, *p* < .001, Cohen’s *d* = 0.74, 95% CI [0.85, 2.26]), and in objects (*t*(57.8) = 3.01, *p* = .003, Cohen’s *d* = 0.51, 95% CI [0.36, 1.79]).

In contrast, the interaction between odour and image valence (*F*(3, 6586.5) = 1.07, *p* =.359, $\eta_{p}^{2}$ < 0.01), and the three-way interaction among odour, image valence and image type (*F*(6, 6586.5) =0.06, *p* =.999, $\eta_{p}^{2}$ < 0.01) was not significant. Table J1 presents the estimates of fixed and random effects from the mixed-effects model of image pleasantness ratings.

**Table J1**

| *Full mixed-effects model estimates for image pleasantness without lemon condition* | | | | | |
| --- | --- | --- | --- | --- | --- |
| Fixed Effects | | | | | |
|  | Est/Beta | SE | 95% CI | *t* | p |
| Intercept | 2.12 | 0.34 | 1.48 – 2.78 | 6.30 | 0.0000000103 |
| Odour  Lavender | 0.32 | 0.18 | -0.03 – 0.66 | 1.78 | 0.0748 |
| Odour  Rose | 0.12 | 0.18 | -0.23 – 0.47 | 0.66 | 0.4957 |
| Odour  Vanilla | 0.33 | 0.18 | -0.02 – 0.67 | 1.84 | 0.0658 |
| Valence  Positive | 1.70 | 0.39 | 0.96 – 2.46 | 4.41 | 0.0000297 |
| Type  Landscape | 2.09 | 0.39 | 1.32 – 2.81 | 5.44 | 0.000000474 |
| Type  Object | -0.30 | 0.39 | -1.05 – 0.44 | -0.78 | 0.4380 |
|  | | | | | |
| Lavender x Positive | -0.12 | 0.25 | -0.61 – 0.37 | -0.48 | 0.6313 |
| Rose x Positive | -0.20 | 0.25 | -0.69 – 0.29 | -0.80 | 0.4239 |
| Vanilla x Positive | -0.25 | 0.25 | -0.74 – 0.24 | -0.98 | 0.3264 |
|  | | | | | |
| Lavender x Landscape | -0.42 | 0.25 | -0.91 – 0.07 | -1.67 | 0.0952 |
| Rose x Landscape | 0.06 | 0.25 | -0.43 – 0.55 | 0.24 | 0.8083 |
| Vanilla x Landscape | -0.12 | 0.25 | -0.61 – 0.37 | 0.462 | 0.6443 |
| Lavender x Object | 0.07 | 0.25 | -0.42- 0.55 | 0.29 | 0.7737 |
| Rose x Object | 0.30 | 0.25 | -0.19 – 0.79 | 1.19 | 0.2345 |
| Vanilla x Object | 0.23 | 0.25 | -0.26 – 0.72 | 0.92 | 0.3603 |
|  | | | | | |
| Positive x Landscape | 2.56 | 0.54 | 1.39 – 3.63 | 4.72 | 0.00000867 |
| Positive x Object | -0.47 | 0.54 | -1.54 – 0.57 | -0.86 | 0.3919 |
|  | | | | | |
| Lavender x Positive x Landscape | 0.01 | 0.35 | -0.68 – 0.70 | 0.03 | 0.9765 |
| Rose x Positive x Landscape | -0.08 | 0.35 | -0.77 – 0.62 | -0.21 | 0.8312 |
| Vanilla x Positive x Landscape | 0.04 | 0.35 | -0.65 – 0.74 | 0.13 | 0.8992 |
| Lavender x Positive x Object | -0.06 | 0.35 | -0.76 – 0.63 | -0.18 | 0.8582 |
| Rose x Positive x Object | 0.03 | 0.35 | -0.66 – 0.73 | 0.10 | 0.9225 |
| Vanilla x Positive x Object | -0.01 | 0.35 | -0.70 – 0.68 | -0.02 | 0.9809 |
|  | | | | | |
| Random Effects | | | | | |
|  | | | Variance | S.D. | |
| Image (Intercept) | | | 0.73 | 0.85 | |
| Participant (Intercept) | | | 1.25 | 1.12 | |
| Residuals | | | 4.42 | 2.10 | |
|  | | | | | |
| Model fit | | | | | |
| R^2^ | | | Marginal | Conditional | |
|  | | | 0.29 | 0.71 | |

*Note.* Model: Ratings ~ Odour * Valence * Type + (1|Participant) + (1|Image). Clean air, neutral and face were considered as baseline categories.

***Ancova model***

After including all the LEOS emotional dimensions (happiness, disgust, nostalgia, thirstiness, relaxation, sensuality, and energy) as covariates in the model, the main effect of image valence on pleasantness ratings remained significant (F(1, 54.06) = 101.26, *p* <.001, $\eta_{p}^{2}$ = 0.65), and the main effect of image type was also significant (F(2, 52.89) = 100.78, *p* <.001, $\eta_{p}^{2}$ = 0.79). However, the main effect of odour was no longer significant (F(3, 1875.91) = 1.23, *p* =.296, $\eta_{p}^{2}$ < 0.01). This indicates that the variance previously attributed to odour overlapped with participants’ emotional responses to the fragrances, such that controlling for LEOS dimensions removed this shared variance rather than demonstrating a casual mechanism of odour effects.

Moreover, subjective levels of nostalgia (F(1, 1769.96) = 5.03, *p* =.025, $\eta_{p}^{2}$ < 0.01), disgust (F(1,1033.08) = 44.32, *p* <.001, $\eta_{p}^{2}$ = 0.04), sensuality (F(1, 1238.76) = 11.39, *p* < .001, $\eta_{p}^{2}$ < 0.01), and energy (F(1, 1913.00) = 4.40, *p* =.036, $\eta_{p}^{2}$ < 0.01) ratings were significantly associated with variance in image pleasantness ratings that covaried with odour-related differences. The other covariates were not significantly associated (*p* > .05), further details are found in Table J2.

Finally, the interaction between odour and image type was significant (F(6, 1899.90) = 2.45, *p* = .002, $\eta_{p}^{2}$ < 0.01), suggesting that odour effects varied across image categories. Exploratory follow-up pairwise comparisons showed that, after accounting of the LEOS covariates in the model, faces were rated significantly more pleasant in the presence of lavender compared to the clean air condition (*t*(1995) = 2.52, *p* = .012, Cohen’s *d* = 0.30, 95% CI [0.05, 0.54]). Moreover, faces were rated significantly less pleasant in the presence of rose (*t*(1987) = -3.37, *p* < .001, Cohen’s *d* = -0.39, 95% CI [-0.63, -0.15]), and vanilla (*t*(1988) = -2.73, *p* = .006, Cohen’s *d* = -0.32, 95% CI [-0.57, -0.08]) compared to the lavender odour condition. Furthermore, none of the odour pairs differed significantly for landscape images and for object images (all *p* >.05).

The interaction between image valence and image type was also significant (F(2, 54.05) = 15.76, *p*<.001, $\eta_{p}^{2}$ = 0.37), indicating that the effect of valence differed across image categories. Exploratory follow-up pairwise comparison showed that, after accounting for the LEOS covariates in the model, positive pictures were rated significantly higher than neutral in landscapes (*t*(165) = 9.70, *p* < .001, Cohen’s *d* = 1.82, 95% CI [1.43, 2.20]), in faces (*t*(169) = 4.16, *p* <.001, Cohen’s *d* = 0.77, 95% CI [0.39, 1.15]), and in objects (*t*(159) = 2.58, *p* = .011, Cohen’s *d* = 0.48, 95% CI [0.10, 0.87]). The interaction between odour and image valence was not significant (F(3, 1899.95) = 1.06, *p*= .365, $\eta_{p}^{2}$ < 0.01), and the three-way interaction among odour, image valence and image type was also not significant (F(6, 1899.95) =0.37, *p*=.899, $\eta_{p}^{2}$ < 0.01). Table J3 presents the fixed and random effects estimates from the mixed-effects model with LEOS covariates for image pleasantness ratings.

**Table J2**

| *Table for mixed-effects ANCOVA model* | | | | | | | | | | |
| --- | --- | --- | --- | --- | --- | --- | --- | --- | --- | --- |
| Variable/covariate | | *df_num_* | | *df_den_* | | *F* | | *p* | |  |
| Odour | | 3 | | 1875.91 | | 1.23 | | .296 | |  |
| Valence | | 1 | | 54.06 | | 101.26 | | <.001* | |  |
| Type | | 2 | | 52.89 | | 100.78 | | <.001* | |  |
| Happiness | | 1 | | 1905.48 | | 1.50 | | .221 | |  |
| Nostalgia | | 1 | | 1769.96 | | 5.03 | | .025* | |  |
| Disgust | | 1 | | 1033.08 | | 44.32 | | <.001* | |  |
| Relaxation | | 1 | | 1909.96 | | 1.09 | | .230 | |  |
| Sensuality | | 1 | | 1238.76 | | 11.39 | | <.001* | |  |
| Thirstiness | | 1 | | 1814.36 | | 2.26 | | .133 | |  |
| Energy | | 1 | | 1913.00 | | 4.40 | | .036* | |  |
| Odour:Valence | 3 | | 1899.95 | | 1.06 | | .365 | |  |  |
| Odour:Type | 6 | | 1899.90 | | 2.45 | | .002* | |  |  |
| Valence:Type | 2 | | 54.05 | | 15.76 | | <.001* | |  |  |
| Odour:Valence:Type | 6 | | 1899.95 | | 0.37 | | .899 | |  |  |

**Table J3**

| *Full mixed-effects model estimates for the LEOS model* | | | | | |
| --- | --- | --- | --- | --- | --- |
| Fixed Effects | | | | | |
|  | Est/Beta | SE | 95% CI | *t* | p |
| Intercept | 0.98 | 0.57 | -0.15– 2.13 | 1.73 | 0.09198 |
| Odour  Lavender | 0.97 | 0.35 | 0.29 – 1.66 | 2.78 | 0.00551 |
| Odour  Rose | 0.05 | 0.35 | -0.64 – 0.74 | 0.15 | 0.88323 |
| Odour  Vanilla | 0.31 | 0.35 | -0.38 – 1.00 | 0.890 | 0.37362 |
| Valence  Positive | 2.16 | 0.48 | 1.16 – 3.11 | 4.48 | 0.0000153 |
| Type  Landscape | 2.66 | 0.48 | 1.71 – 3.62 | 5.515 | 0.000000167 |
| Type  Object | 0.16 | 0.48 | -0.81 – 1.11 | 0.333 | 0.73957 |
| Happiness | 0.04 | 0.03 | -0.02– 0.10 | 1.23 | 0.22077 |
| Nostalgia | 0.06 | 0.03 | 0.01 – 0.11 | 2.24 | 0.02500 |
| Disgust | -0.22 | 0.03 | -0.28 – -0.15 | -6.66 | 0.0000000000451 |
| Relaxation | 0.03 | 0.03 | -0.03 – 0.09 | 1.05 | 0.29562 |
| Sensuality | 0.13 | 0.04 | 0.05 – 0.21 | 3.38 | 0.00076 |
| Thirstiness | 0.03 | 0.02 | -0.01 – 0.08 | 1.51 | 0.13258 |
| Energy | -0.06 | 0.03 | -0.12 – -0.01 | -2.10 | 0.03612 |
|  | | | | | |
| Lavender x Positive | -0.64 | 0.48 | -1.58 – 0.29 | -1.35 | 0.17586 |
| Rose x Positive | -0.52 | 0.48 | -1.45 – 0.42 | -1.08 | 0.27926 |
| Vanilla x Positive | -0.74 | 0.48 | -1.68 – 0.19 | -1.56 | 0.11815 |
|  | | | | | |
| Lavender x Landscape | -1.35 | 0.48 | -2.29 – -0.42 | -2.84 | 0.00453 |
| Rose x Landscape | -0.18 | 0.48 | -1.11 – 0.75 | -0.38 | 0.70671 |
| Vanilla x Landscape | -0.41 | 0.48 | -1.34 – 0.53 | -0.86 | 0.39161 |
| Lavender x Object | -0.29 | 0.48 | -1.23 – 0.64 | -0.62 | 0.53611 |
| Rose x Object | 0.34 | 0.48 | -0.59 – 1.27 | 0.72 | 0.47463 |
| Vanilla x Object | 0.26 | 0.48 | -0.67 – 1.19 | 0.54 | 0.58632 |
|  | | | | | |
| Positive x Landscape | 1.80 | 0.68 | 0.33 – 3.42 | 2.64 | 0.00939 |
| Positive x Object | -0.78 | 0.68 | -2.12 – 0.61 | -1.14 | 0.25504 |
|  | | | | | |
| Lavender x Positive x Landscape | 0.85 | 0.67 | -0.47 – 2.17 | 1.26 | 0.20793 |
| Rose x Positive x Landscape | -0.52 | 0.67 | -0.81 – 1.84 | 0.77 | 0.44407 |
| Vanilla x Positive x Landscape | 0.58 | 0.67 | -0.74 – 1.90 | 0.86 | 0.39209 |
| Lavender x Positive x Object | 0.09 | 0.67 | -1.23 – 1.41 | 0.13 | 0.89898 |
| Rose x Positive x Object | 0.32 | 0.67 | -1.00– 1.64 | 0.48 | 0.63500 |
| Vanilla x Positive x Object | 0.23 | 0.67 | -1.09 – 1.55 | 0.34 | 0.73362 |
|  | | | | | |
| Random Effects | | | | | |
|  | | | Variance | S.D. | |
| Image (Intercept) | | | 0.83 | 0.91 | |
| Participant (Intercept) | | | 1.84 | 1.36 | |
| Residuals | | | 4.76 | 2.18 | |
|  | | | | | |
| Model fit | | | | | |
| R^2^ | | | Marginal | Conditional | |
|  | | | 0.28 | 0.72 | |

*Note.* Model: Ratings ~ Odour * Valence * Type + Happiness + Nostalgia + Disgust + Relaxation + Sensuality + Thirstiness + Energy+ (1|Participant) + (1|Image). Clean air, neutral and face were considered as baseline categories.

**Reference list**

Champely, S. (2020). *pwr: Basic Functions for Power Analysis*. In CRAN: Package pwr

Lang, P. J., Bradley, M. M., & Cuthbert, B. N. (2008). *International affective picture system (IAPS): Affective ratings of pictures and instruction manual.* (Technical Report A-8., Issue.

Ma, D. S., Correll, J., Wittenbrink, B., Ma, D. S., Correll, J., & Wittenbrink, B. (2015). The Chicago face database: A free stimulus set of faces and norming data. *Behavior Research Methods 2015 47:4*, *47*(4). <https://doi.org/10.3758/s13428-014-0532-5>
